# Concurrent Reduction and Stabilization of Graphene Oxide Dispersion by Silk-Inspired Polymer

**DOI:** 10.1021/acsapm.3c00353

**Published:** 2023-06-15

**Authors:** Zoren Valmonte, Zeyad Baker, Jianna Loor, Amrita Sarkar

**Affiliations:** † Department of Chemistry and Biochemistry, Montclair State University (MSU), Montclair, New Jersey 07043, United States; ‡ Department of Biology, Montclair State University (MSU), Montclair, New Jersey 07043, United States

**Keywords:** silk-inspired polymer, microwave-assisted Diels−Alder, green reducing agent, reduced graphene oxide, aquatic dispersion stability

## Abstract

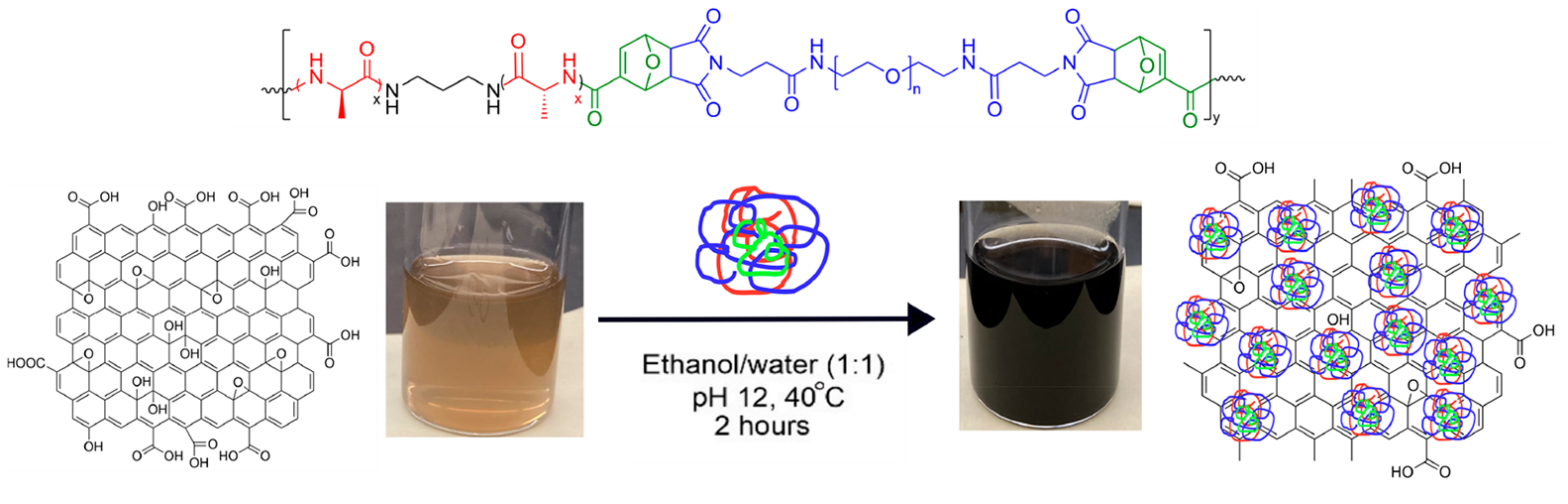

Silk, a popular biomaterial, is used as a greener alternative of toxic reducing agent in biocompatible graphene synthesis. However, silk often forms gel uncontrollably due to its heavy-chain molecular weight and faces significant challenges in the reduction, stabilization, and dispersion process of graphene. In this contribution, we report a rapid chemical synthesis approach for a low-molecular-weight silk-inspired polymer via ring-opening and microwave-assisted Diels–Alder-aided step-growth polymerizations. This synthetic polymer with periodic sequences of hydrophilic and hydrophobic moieties not only reduces graphene oxide efficiently but also enhances the dispersibility of hydrophobic reduced graphene oxide in aqueous media.

Reduced graphene oxide (rGO) serves as a key material in a variety of applications including energy storage, composite material fabrication, tissue engineering, drug delivery, biosensors, and bioimaging devices, due to its exceptional thermal, electrical, mechanical, and optical properties.^1−4^ Large-scale rGO production relies on a solution-based chemical reduction method of exfoliated graphene oxide (GO) primarily.^4^ A number of conventional low-cost reducing agents (e.g., hydrazine and sodium borohydride) have been reported to produce rGO from GO successfully.^2,3^ However, their toxic and hazardous nature not only limits their applications but often requires the addition of a capping agent (e.g., polymer and surfactant) to prevent precipitation of hydrophobic rGO.^1,4−6^ Thus, there is a constant effort in finding greener alternatives with the dual properties of reduction and stabilization that can be used in the fabrication of biocompatible rGO. Over the decades, significant progresses have been made toward rGO synthesis using natural products, such as vitamin C, green tea, glycine, β-lactoglobulin, and bovin serum albumin.^5,7−10^ Despite success, the majority of them demonstrated limited scalability and water solubility, prolonged reaction time, and relatively high cost compared to their synthetic counterparts.^4,6,7,11^ Likewise, a few low-cost synthetic polymers and poly(amino acids) [e.g., pluronic, poly(ethylene glycol) (PEG), poly(sodium-4-styrenesulfonate), poly(l-lysine)] have been explored for enhancing the dispersibility of rGO in water.^6,12−14^ However, they were not reported in reducing GO alone; hydrazine monohydrate or sodium borohydride performed the reduction.

Recently, natural biopolymer silk is recognized as a promising and cost-effective approach for graphene and graphene composite biomaterial synthesis.^6,11,15,16^ For instance, Wu and co-workers^6^ used regenerated silk fibroin (SF) in the fabrication of rGO/gold nanocomposite, exploiting its reduction and stabilization properties. Kaplan and co-workers^11^ demonstrated a remarkable pathway of mass-scale graphene production using silk nanofibers. Their approach led to the formation of excellent aqueous dispersion of graphene with higher concentration (>8 mg mL^–1^). Later Park et al.^16^ reported the fabrication of printable bioink through the reduction of GO by chemically modified SF, which showed a high degree of stability in a hydrophilic environment with no trace of sedimentation. Silk protein is a prospective biomaterial with its remarkable mechanical properties and excellent biocompatibility.^17−19^ It owns a large repetitive core domain consisting of a periodic sequence of hydrophobic alanine and hydrophilic glycine-rich alternating blocks.^17,18,20^ Silk protein typically reduces GO through its cysteine, tyrosine, and glycine residues.^6,15^ Upon self-assembly, hydrogen-bonded β-sheet nanocrystallites form that favor hydrophobic interaction, whereas the hydrophilic glycine-rich block forms an amorphous matrix that facilitates hydrophilic interaction. Due to this unique structure, silk is adsorbed on the hydrophobic graphene surface and prevents it from aggregating in an aqueous environment. However, silk often tends to form gel during the exfoliation process of graphene due to its heavy-chain molecular weight (370 kDa)^21^ and is thus found to be unsuitable for mass-scale graphene production.^11^ We hypothesize that a silk-inspired man-made polymer having balanced hydrophobic/hydrophilic interaction and lower molecular weight forms rGO without forming gel. We are also motivated by the well-established fact that scalability, structural stability, and chemical diversity of the synthetic polymer are advantages over their natural counterparts.^22^ Inspired by these, here we put forward a robust chemical synthesis strategy for a low-molecular-weight silk-inspired polymer. This synthetic polymer reduces GO alone without assistance of a conventional reducing agent and forms a stable aquatic dispersion of rGO (Scheme 1).

**Scheme 1:**
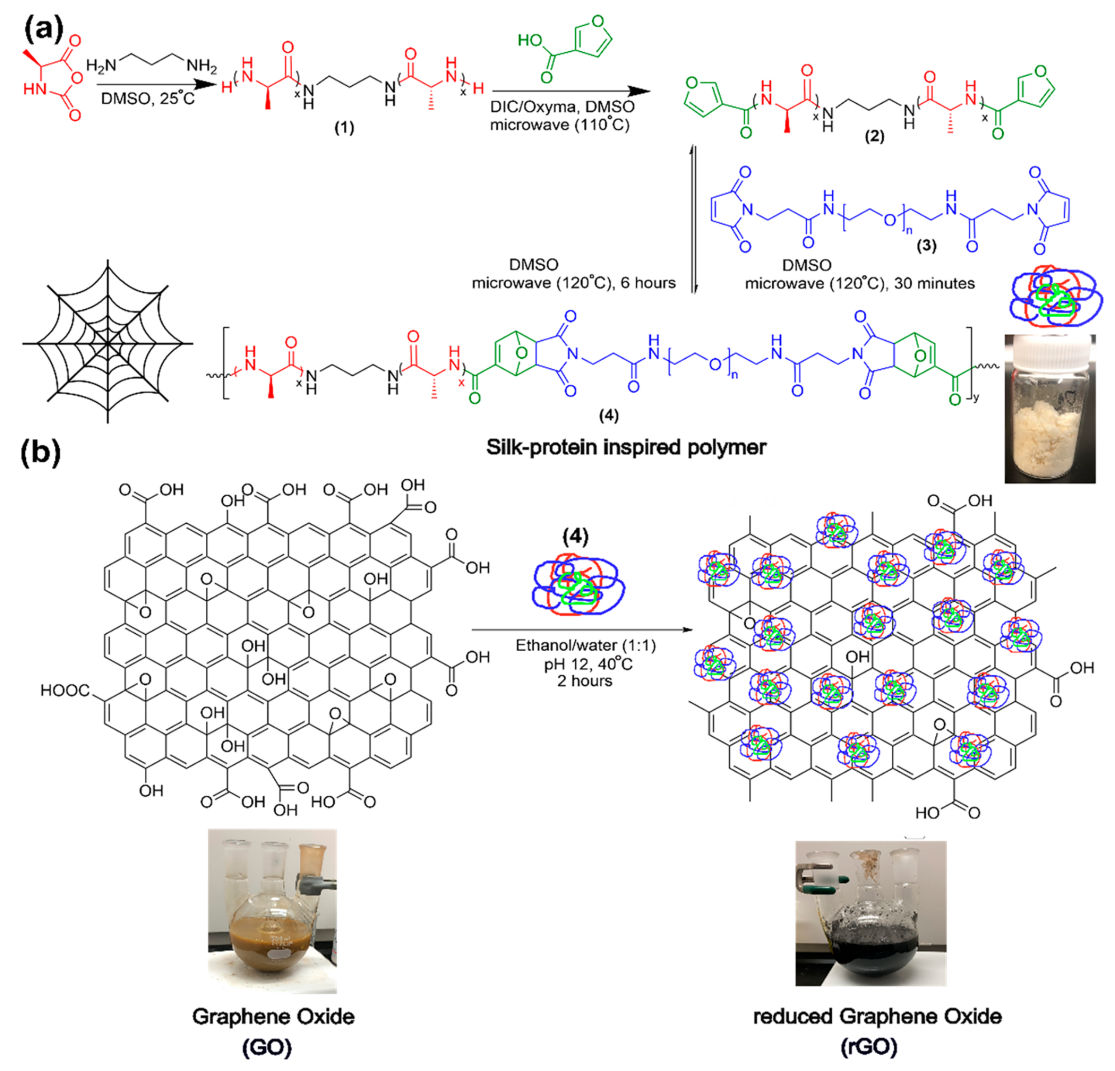
Schematic Depiction for the Synthesis of Silk-Inspired Polymer (a) and Its Use in rGO Preparation (b)

To this end, silk-inspired (AB)_*n*_ polymer (**4**) was synthesized using premade end-group-functionalized triblock oligo(alanine)propandiamine (**2**) and poly(ethylene glycol)bismaleimide [PEG(maleimide)_2_] (**3**) (Scheme 1). **2** was selected as block A, synthesized via ring-opening polymerization (ROP) of *N*-carboxyanhydride alanine (NCA-ALA) and the initiator propane-1,3-diamine followed by end-group functionalization to a furan derivative by diisopropylcarbodiimide/ethyl cyano(hydroxyimino)acetate coupling chemistry (Schemes S1 and S2).^20,23^ We chose **3** as a hydrophilic and amorphous rubbery matrix B, a replacement of glycine due to its commercial availability and biological tolerance. Both blocks A and B were conjugated via microwave-assisted furan–maleimide Diels–Alder (DA)-based step-growth polymerization (Schemes 1 and S3). The synthetic procedure, reaction efficiency, and related discussions are described in Section 1 in the Supporting Information. Resultant homopolymers and silk-inspired polymer were characterized by ^1^H NMR, matrix-assisted laser desorption/ionization time-of-flight (MALDI-TOF) mass spectrometry (MS), and gel permeation chromatography (GPC) (Figures 1a,b and S1–S4). The complete disappearance of aromatic protons of furan in the range of 6.5–7.5 ppm and the simultaneous appearance of new proton signals from DA adducts at 3.14, 5.5, and 6.99 ppm were noticed in a representative ^1^H NMR spectrum (Figure 1a). This result indicates that a successful DA reaction^24^ occurred that resulted in forming silk-inspired polymer **4** in 30 min only, facilitated by establishing a synergistic effect^25^ between the applied microwave irradiation and increased reactivity of predefined end-functionalized homopolymers **2** and **3**. GPC profiles for the synthesized polymers demonstrated clear shifts to lower retention time or higher molecular weights compared to their homopolymer precursors (Figures 1b and S4). GPC analysis for the synthesized silk-inspired polymers exhibited number-average (*M*_n_) and weight-average (*M*_w_) molecular weights in the moderate ranges of 5.4–15.7 and 11–67 kg mol^–1^, respectively (Table S1). We attribute the lower molar masses to the probable stoichiometric imbalance or trace presence of impurities in the polydisperse homopolymers A and B, a common characteristic of traditional homogeneous step-growth polymerization.^26^ This could be improved using uniform homopolymer oligo(alanine) instead of a polydisperse one, which would be obtained by changing the reaction strategy to solid-phase peptide synthesis from ROP.^20^ While the primary focus of the current work was to employ a forward DA reaction to click furan- and maleimide-functionalized homopolymers, we briefly investigated its thermoreversibility. During the extended reaction at high temperature, retro DA dominates; thus, furan–maleimide linkages cleaved, which led to the formation of individual blocks A and B with free furan and maleimide functionalities. However, DA re-formation was not observed upon cooling and subsequent heating of the reaction mixture. This observation is consistent with the other literature^27^ where the limited re-formation of DA adducts was reported and attributed to the sacrificial cross-linking nature of the DA moiety.

**Figure 1:**
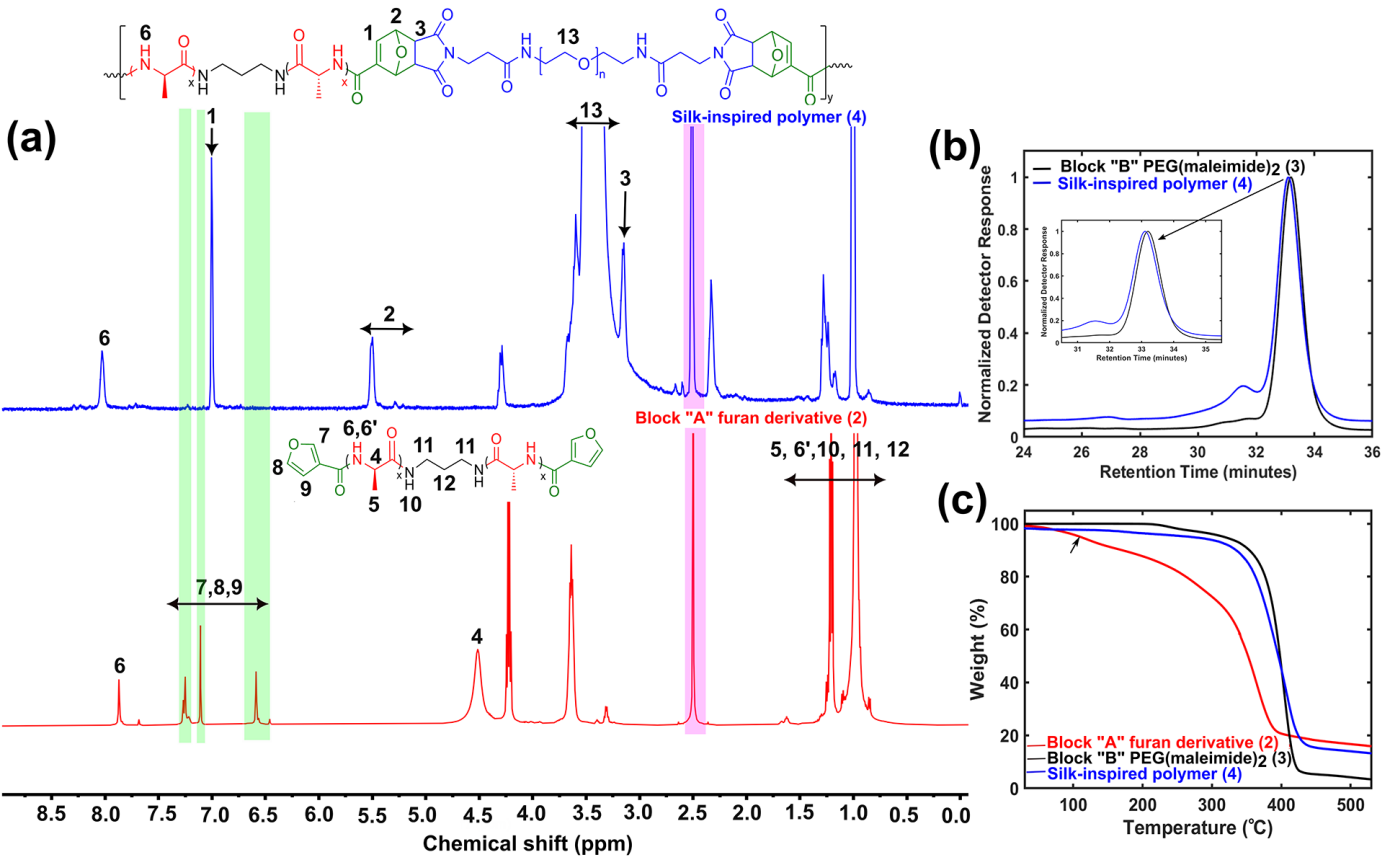
Characterization of silk-inspired polymer **4**: (a) ^1^H NMR spectra showing the complete disappearance of aromatic protons of furan (green highlighted) and the appearance of new signals from the DA adduct, suggesting a successful DA reaction. Both spectra were obtained in DMSO-*d*_6_, highlighted in pink. (b) GPC analysis showing a clear peak shift to lower retention time, with *M*_n_ and *M*_w_ values of 5.8 and 12.5 g mol^–1^, respectively, along with a *Đ* value of 2.2. (c) TGA profile exhibiting the initial weight loss (5%) for block A at 108 °C (shown by arrow), whereas for block B and polymer **4**, degradation started above 300 °C.

The thermal properties of the synthesized polymer and precursor homopolymers were evaluated next. Thermogravimetric analysis (TGA) for polymer **4** showed an increased thermal stability whose degradation started above 330 °C, whereas the peptide precursor A demonstrated an initial degradation at 108 °C (possibly due to the removal of water molecules) and 10% decomposition at ∼167 °C (Figure 1c and Table S3). This significant increment in thermal stability is attributed to the linkage between blocks A and B through the DA adduct. However, the thermal stability of polymer **4** was found to be somewhat lower than that of B block **3**, possibly due to the instability of the DA adduct at higher temperature. Detailed thermal analysis is discussed in Section 2 and Figure S5.

In order to verify the secondary structure of the synthesized polymers, we performed a combination of characterization techniques including Fourier transform infrared (FTIR), X-ray diffraction (XRD), and transmission electron microscopy (TEM) (Figure 2). FTIR for oligo(alanine)propandiamine (**1**) reveals a strong peak at 1626 cm^–1^, which is a signature peak for the antiparallel β-sheet structure observed in the natural spider silk.^17,20,23,28^ The same signal was found in the silk-inspired polymer **4** (Figure 2a), which indicates that the formation of an antiparallel β-sheet structure was unaffected during the conjugation of PEG to oligo(alanine) via a microwave-assisted DA reaction (Figure S6). In addition to that, the appearance of a less pronounced peak at 1656 cm^–1^ corresponds to the presence of a random-coil or helical secondary structure.^20,23^ A large and intense band was found at 1706 cm^–1^, which originated from the amide carbonyl group of the soft segment **3**. Furthermore, XRD (Figure 2b) for the synthesized polymer **4** confirmed the retention of well-defined diffraction peaks with *d* spacings of 5.30 and 4.38 Å, consistent with the antiparallel β-sheet structure found in natural silk.^17,20,23^ It is important to note that formation of the β-sheet structure was not affected by the presence of a non-natural DA adduct. Moreover, XRD supports the successful occurrence of DA, demonstrating the complete disappearance of the furan crystalline peaks in polymer **4** (Figure S7). Next, we conducted TEM/cryo-TEM; we found that sequence-controlled silk-inspired polymer **4** self-assembles into spherical micellelike nanostructures^29,30^ in aqueous solution with an average diameter of 49.1 ± 10.4 nm (Figure 2c,d). However, the drop-cast sample exhibited fiberlike structures (Figure 2e) with an average width of 9.1 ± 1.3 nm. The fibers probably originated from the stacking of β sheets in a fibril format, which is induced by a drying phenomenon during electron microscopy sample preparation.^20,28,31^ These data altogether confirm the successful synthesis of a low-molecular-weight polymer that shows self-assembly behavior similar to natural silk.

**Figure 2:**
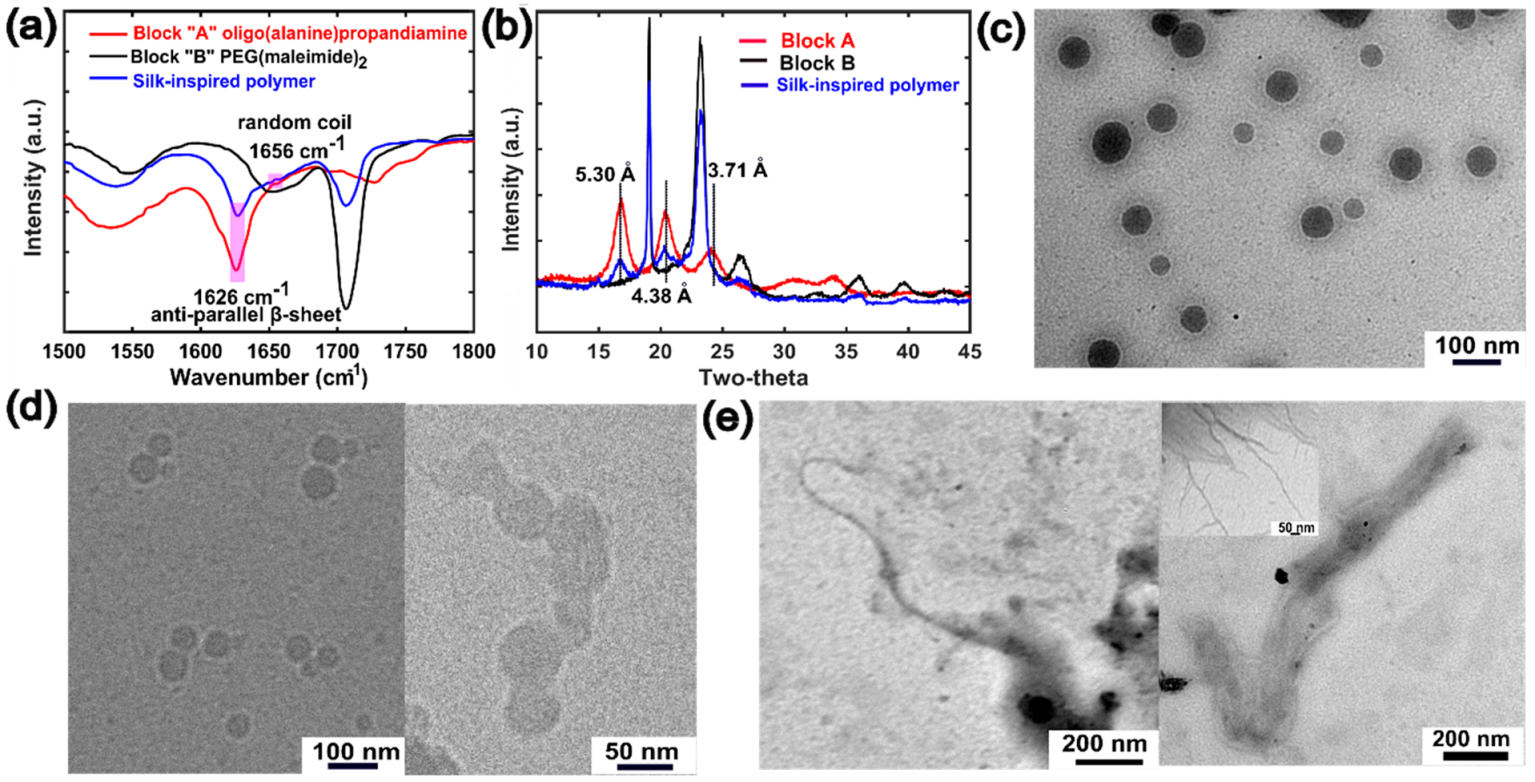
Self-assembly of silk-inspired polymer **4**: FTIR (a) and XRD (b) of the lyophilized polymer sample indicate the formation of an antiparallel β-sheet structure. TEM (c) and cryo-TEM (d) of the polymer aqueous solution show the formation of spherical micelles. TEM image (e) of the drop-cast aliquot of an incubated polymer solution exhibits a bundle of nanofibers stacked together.

Once the formation of a silk-like nanostructure was confirmed in the synthesized polymer **4**, we employed it in the reduction of GO. To compare the reduction efficiency, a control reaction was conducted simultaneously using GO and a common reducing agent, hydrazine monohydrate. Reduction reaction details are described in Section 3 and Figure S8. Upon complete reduction, hydrazine-reduced GO aggregates and precipitates immediately as it becomes hydrophobic. This is due to the removal of oxygenated functional groups from GO and the dominance of π–π stacking between the rGO sheets. Thus, it requires the addition of a capping agent to avoid the formation of irreversible graphene agglomeration.^5,8^ In contrast, black powder rGO (the initial GO solution was dark yellow) obtained by a polymer-assisted reduction process was dispersed in the reaction flask for 1 day (Figure S8). This observation suggests that the reduction of GO and the adsorption capping of polymer **4** on the rGO surface took place simultaneously. Natural silk protein generally reduces GO through its building components including cysteine, tyrosine, and glycine residues.^6,15^ Amine and hydroxyl functional groups of these amino acids were proposed to attack the oxygenated functional groups of GO (e.g., epoxy and hydroxyl) via a nucleophilic substitution reaction and led to the formation of rGO.^7,15^ The detailed reduction mechanism of GO via our proposed polyamide **4** is not clearly understood at this moment. We hypothesize that the reduction might happen through amide and imide groups of polymer **4** under the employed basic condition. On the other hand, the hydrophobic A block oligo(alanine) moiety acts as a capping agent by adsorbing on the rGO surface via hydrophobic β-sheet interaction.^32^ Likewise, the B block hydrophilic PEG chain extends in aqueous media, rendering hydrophilicity onto the alanine-covered rGO surface, and helps to disperse it in water,^12^ thus endowing the rGO particles with a prolonged 3 days of aquatic stability (Figure S9). TEM images (Figure S10) of nonaggregating rGO sheets further confirm the stability of the polymer-coated rGO dispersion. It is noteworthy to mention that no sign of gel formation was noticed during the silk-inspired polymer-assisted reduction process. To confirm the successful reduction, several characterizations were performed after repetitive washing of rGO to ensure complete removal of the free unbound polymer from it. FTIR (Figure 3a) for polymer-assisted rGO showed the complete disappearance of the carbonyl (C=O) stretching vibration peak at 1720 cm^–1^.^33^ Another characteristic broad absorption band intensity decreases at 3000–3500 cm^–1^, which is primarily assigned to the hydroxyl (−OH) functional group. These observations matched the hydrazine reduction profile, which suggests that most oxygenated functional groups were eliminated by the polymer-assisted reduction method. However, a narrow peak responsible for the −NH stretching vibration was visible at 3271 cm^–1^, which suggests a silk-inspired polymer attached to the rGO surface.^6^ Likewise, a new peak appeared at 1540 cm^–1^, which is assigned to the sp^2^ carbon atom stretching vibration. Typically, this vibration appears at ∼1572 cm^–1^ when rGO regains its conjugated structure.^8^ However, we noticed the shifted value due to the dominance of the random-coil secondary structure of the silk-inspired polymer adsorbed onto the conjugated rGO surface. Furthermore, there is FTIR evidence of a sharp peak at 1628 cm^–1^ along shoulder peaks at 1638, 1654, and 1693 cm^–1^, which indicates the coexistence of β-sheet, amorphous random-coil, and β-turn secondary structures in polymer **4** adsorbed onto the hydrophobic rGO surface. This observation is in good agreement with the molecular dynamics simulation and supported experimental observations reported by Tsukruk and co-workers.^34^ They demonstrated their findings of a gradual decrease in the β-sheet content and an increase in the random-coil structure while silk protein adsorbed onto the graphene surface.^34^ The reduction efficiency was further investigated by UV–vis absorption. GO exhibits two strong absorption maxima at 226 and 315 nm, which are ascribed to π–π* and n−π* transitions.^6,7,15^ We observed that the first peak shifted to 270 nm, with complete vanishing of the latter peak at 315 nm in both rGO samples (Figure 3b). This shift was explained in terms of the reduction of GO along the restoration of the electronic conjugation or sp^2^ network.^3,5−7^ Additional XRD measurements (Figure 3c) showed comparable diffraction profiles for both rGOs. Almost the disappearance of the sharp diffraction peak at 10.89° and the appearance of a peak with 2θ of 22.89° indicate the successful elimination of oxygenated functional groups from GO during both reduction processes.^1,3,7^ This new broadened diffraction peak in polymer-assisted rGO is found to be somewhat close to the (002) diffraction peak for hexagonal graphite (2θ = 26.48° and *d* spacing of 3.36 Å) with an interlayer *d* spacing of 3.88 Å. In addition to that, several other peaks were found in the 2θ range of 30–40° for polymer-assisted rGO. This is probably due to intercalation of the polymer chain into the rGO layer via hydrogen bonding and β-sheet hydrophobic interactions. To evaluate the thermal stability of rGO obtained by a polymer-assisted reduction process, TGA was conducted (Figure S11). TGA showed that mass loss started at an onset temperature of 335 °C, which corresponds to the removal of the majority oxygenated functional groups along with the decomposition of residual bound polymer. We also analyzed the rGO morphologies by electron microscopy, including scanning electron microscopy (SEM) and TEM. SEM showed agglomeration of closely spaced, separated thinly crumpled sheets suggestive of the nature of rGO,^1,2^ in contrast to GO, which showed a stacked configuration (Figure 3d–f). The featureless TEM image and unresolved selected-area electron diffraction (SAED) pattern (inset) for GO depict an amorphous nature (Figures 3g and S12). This regains its crystalline state upon reduction, as is evident by the reestablishing of 6-fold symmetry in the SAED pattern of rGO (Figure 3h,i). This rGO appeared transparent and folded over on its edges (Figure S13), suggestive of the nature of a single graphene sheet.^35^ The corresponding SAED yields bright spots or well-defined 6-fold symmetry with an arc of weaker intensity (Figure 3i and S13), which is possibly diffractions from the bounded silk-inspired polymer. The Brunauer–Emmett–Teller (BET) surface area of rGO obtained by this developed reduction method was found to be 217.62 m^2^ g^–1^, low compared to the theoretical value for isolated graphene sheets but consistent with the values for rGO published in other literature (Figure S14 and Table S4). We attribute the lower value to the attached polymer on rGO, which possibly covers the surface of rGO and leads into the inaccessible surface. Furthermore, the pore volume distribution profile for the same rGO showed a mesoporous structure with a maximum pore volume for the pore diameter of 3.75 nm (Figure S15). All of these results suggest that the oxygenated functional groups in GO have been removed efficiently by our designed silk-inspired polymer, thus providing a green reduction strategy for preparing a conjugated graphene network. The combination of two components, including a periodic sequence of oligo(alanine) and PEG blocks in the synthetic design of a silk-inspired polymer and their favorable hydrophobic/hydrophilic interactions in the dual reduction and stabilization of rGO dispersion, may induce a synergistic effect,^25,36^ which provides nonaggregating rGO with good application prospects (Figures S16 and S17).

**Figure 3:**
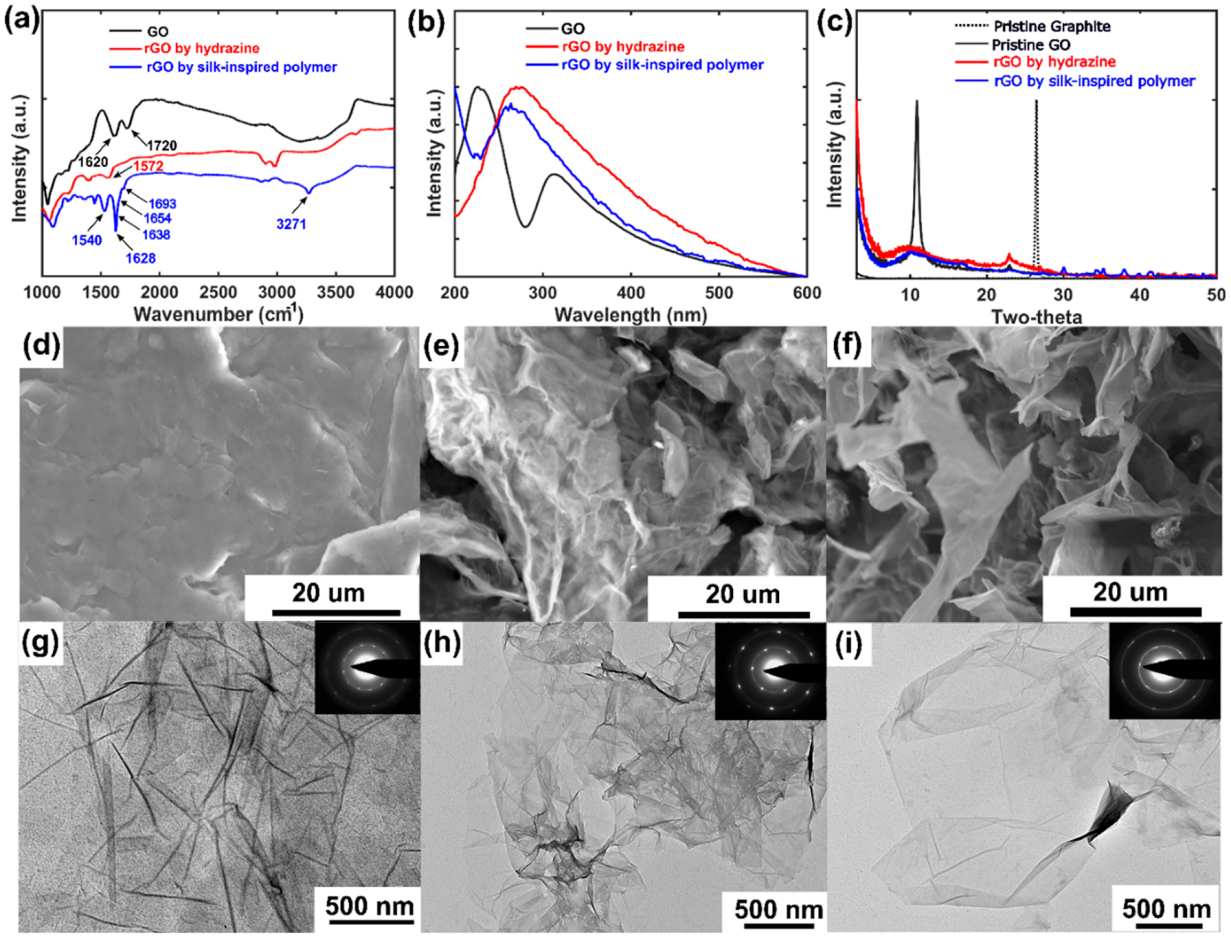
FTIR (a), UV–vis (b), and XRD (c) characterizations of GO and rGO. SEM images of GO (d) and rGO (e and f) showing the morphology change after reduction. Same characterization by TEM along with SAED (inset). TEM images of GO showing an amorphous feature (g) that regains its crystallinity upon reduction by hydrazine monohydrate (h) and polymer **4** (i), respectively.

In summary, we developed a mild reduction strategy to prepare rGO using a chemically synthesized silk-inspired biocompatible polymer, which has great potential for acting as an alternative to the toxic reducing agent hydrazine monohydrate. The polymer design strategy used in this work utilizes a combination of ring-opening and microwave-assisted DA-aided step-growth polymerizations. Controlled conjugation of a crystalline β sheet forming alanine and a hydrophilic PEG moiety in the designed polymer facilitates strong hydrophobic–hydrophilic interactions when attached to the hydrophobic rGO surface. As a result, this designed polymer not only acts as a green reducing agent but also serves as a stabilizer for hydrophobic graphene in an aqueous environment. This unique low-molecular-weight silk-inspired polymer design shows no gel formation and thus may prove useful as a natural silk protein alternative in the rGO synthesis. We believe our straightforward approach paves the way for the design and synthesis of a bioinspired polymer to augment the mass production of rGO-based biocompatible materials.

## Supporting Information Available

Experimental methods (silk-inspired polymer synthesis, reduction strategies, etc.), MALDI-TOF MS, ^1^H NMR, GPC, thermal analysis (TGA and differential scanning calorimetry), FTIR, XRD, TEM (SAED), field-emission SEM (cross section and top view), and BET analysis of the silk-inspired polymer and resultant rGO (PDF)
