# Supplementary material for: Concurrent Reduction and Stabilization of Graphene Oxide Dispersion by Silk-Inspired Polymer
Source: ACS Appl Polym Mater. 2023 Jun 15;5(7):4621–7. doi: 10.1021/acsapm.3c00353 (PMC10353489; doi:10.1021/acsapm.3c00353)
Supplement: Supplementary file 1 — ap3c00353_si_001.pdf [file ap3c00353_si_001.pdf]

Supporting Information

Concurrent Reduction and Stabilization of Graphene Oxide Dispersion by Silk-Inspired Polymer

Zoren Valmonte,[a] Zeyad Baker,[a] Jianna Loor,[b] Amrita Sarkar [a*]
[a]*Department of Chemistry & Biochemistry, Montclair State University, Montclair, NJ 07043, USA.*
[b]*Department of Biology, Montclair State University, Montclair, NJ 07043, USA.*
**Corresponding author: sarkara@montclair.edu*

Materials

Alanine N-carboxyanhydride (Ala NCA) or (S)-4-methyl-2,5-oxazolidionedione (CAS No. 2224-52-4) was purchased from LinkChem Co., Ltd. (Shanghai, China) and used as received. Dimethyl sulfoxide (DMSO, LC-MS grade, 99.9%, Thermo Fisher), N, N-dimethyl formamide (DMF, Certified ACS, 99.8%, Fisher Chemical), absolute ethanol (200 proof, 99.5%, Spectrum™ Chemical), propane-1,3-diamine (99%, Thermo Fisher), 3-furan carboxylic acid (98%, Sigma Aldrich), Ethyl cyano(hydroxyimino)acetate (oxyma, Novabiochem), N,N'-diisopropylcarbodiimide (DIC, 99%, Alfa Aesar), diethyl ether (BHT stabilized, Fisher), PEG (maleimide)$_2$ (average M_n=2,000, and 5,000 g/mol, Jenkem Technology), were used as received. The solvents were dried at room temperature by storing over 50% w/v of molecular sieves (3Å, Alfa Aesar). For graphene derivative synthesis commercial graphite flakes (325 mesh, 99.8%, Thermo Scientific™), sulfuric acid (99.999%, Sigma Aldrich), sodium nitrate (99%, Sigma Aldrich), potassium permanganate (ACS Reagent grade, 99%, Sigma Aldrich), hydrogen peroxide (30%, Sigma Aldrich), hydrochloric acid (ACS Reagent, 37%, Sigma Aldrich), hydrazine monohydrate (reagent grade, 98%, Sigma Aldrich) were commercially purchased, stored at ambient conditions, and used without further purification.

Characterization

Synthesized oligopeptides were characterized by Bruker Autoflex Speed MALDI/TOF mass spectroscopy equipped with a nitrogen smart beam laser (λ = 337 nm, 150 µJ, 3 ns) at a pulse rate of 50 to 100 Hz. Ions were accelerated with a pulsed ion extraction (PIE) by an acceleration voltage of 25KV. The analyzer was operated in reflector mode and the ions were detected using

a micro channel plate detector. Samples were dissolved in DMF or DMSO (1 mg/mL), mixed with α-cyano-4-hydroxycinnamic acid (CHCA), and deposited on a MTP Anchor Chip^{TM} 384 BC target plate.

All NMR experiments were performed at 500 MHz Bruker Avance III HD NMR spectrometer equipped with 5 mm H/F-11B selective probe. All spectra were recorded in DMSO-d_6. The number of scans was set to 16 for Furan Derivative, and to 256 for silk-inspired polymer.

Polymers molecular weights were determined by a Waters Empower gel permeation chromatography (GPC) system equipped with a 717 plus autosampler, a 1525 HPLC pump, and a 2414 refractive index (RI) detector. Columns of PLgel 5 µm guad, PLgel 10 µm MIXED-B and PLgel 5 µm MIXED-C were set at temperature of 50 °C. The measurements were carried out with the sample concentrations of 5 mg/mL eluted by DMF containing 0.1 wt% lithium bromide with a flow rate of 0.5 mL/min. Polymers were dispersed in 1 mL mobile phase and set at 40 °C overnight to fully dissolve followed by slow filtration through 0.2 µm PTFE syringe filter just prior to injection. The number-average (M_n) and weight-average (M_w) molecular weights and dispersity (Đ) were estimated using polymethyl methacrylate (PMMA) standards (Agilent Technologies).

Thermal analyses of the synthesized polymers were performed with a Differential Scanning Calorimeter TA Instrument Discovery DSC in a temperature range of 25 to 300 ºC at a heating rate of 10 ºC min^{-1} under a nitrogen flow of 60 mL min^{-1}. The glass transition temperature (T_g) was determined from the second heating trace and is reported as the midpoint of the thermal transition. Thermal degradation of the polymers was investigated by thermogravimetric analysis (TGA) performed with a TA Instruments Discovery TGA. Measurements were conducted from 25 to 600 ºC at a rate of 10 ºC min^{-1} in a nitrogen flow of 60 mL min^{-1}.

Room temperature Fourier transform infrared (FTIR) spectra of the lyophilized polymers and graphene samples were carried out in a PerkinElmer Spectrum Two FT-IR spectrometer using attenuated total reflectance (ATR). Measurements were carried out in the range of 3000-650 cm^{-1} with a scan number of 32 and resolution of 8 cm^{-1}.

UV-Vis absorption spectra for GO and rGO samples were recorded on an Evolution 300 UV-vis spectrophotometer. The suspension of GO and rGO samples in ethanol/water (50:50) mixtures were measured after 10 minutes sonication. The ethanol/water (50:50) mixture was used as reference.

Diffraction patterns were collected on a Rigaku MiniFlex 6G Powder diffractometer equipped with a 600W Cu Target X-ray source (wavelength λ = 1.5406 Å). XRD patterns were collected in the range of scattering angles 2θ of 3°–90° (for graphite samples) and 10-60 º (for polymers) and a scanning rate of 10º min^{-1}. Data analysis was performed using software Rigaku Smart Studio II. Phase identifications were performed referencing to the crystallography open database (COD).

Top-view SEM images of GO, and rGO were acquired using a Hitachi S-3400N SEM. Samples were mounted on aluminum stub using carbon adhesive and imaged normal to the planar surface. An acceleration voltage of 15 keV and secondary electron detector were used. Working distance was maintained at ~6-10 mm.

Transmission Electron Microscopy (TEM) images along the selected area electron diffraction (SAED) patterns for polymer and graphene samples were acquired using a Hitachi H-7500 tungsten/LaB6TEM operated at 120 kV. Polymer samples were prepared by dissolving lyophilized polymer in the water/ethanol (50:50) mixture with the concentration of 1 mg/mL followed by incubation at 27 ºC for a day. The resultant solution (one drop) was placed on a carbon coated copper grid (Electron Microscopy Sciences, USA), and then placed on a piece of filter paper to remove excess solvent, and air dried before imaging. The sample was not stained before imaging. Graphene samples were prepared in the same fashion, except the sample concentration was maintained at 1 mg/mL and sonicated for 5 minutes instead of incubation. Cryogenic transmission electron microscopy (cryo-TEM) vitrified polymer samples were prepared placing the polymer solutions on holey carbon grids on a controlled environment vitrification system, which contained saturated water vapor to prevent evaporation of water from the polymer solution. PELCo easyGlow™ was used to give the Quantifoil R2/2 Cu 200 grids a negative charge to allow easier application of the polymer solution. Next, the

sample was immediately dropped into liquid ethane at a temperature of -180 ºC. Grids with vitrified sample solutions were maintained at liquid nitrogen temperature and then cryo-transferred to the microscope. Imaging was performed on a 200 kV Talos Arctica cryo TEM (Thermo Fischer) with K2 direct detector (Gatan) controlled by Serial EM software.

The Brunauer-Emmett-Teller (BET) surface area and the Barrett-Joyner-Halenda (BJH) pore size distributions for the rGO powder were measured by nitrogen adsorption using surface area and porosity analyzer (Quantachrome Nova 2000e). Prior to the measurement the sample was degassed at 150 ºC for 8 hours under vacuum conditions to remove impurities and water.

Section 1. Polymer Synthesis

Scheme S1. Ring Opening Polymerization (ROP) of N-carboxyanhydride of Alanine (NCA-ALA).

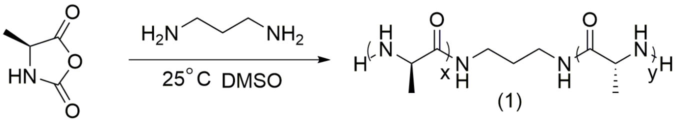

Scheme S1. Synthesis of oligo(alanine)propandiamine **(1)**.

The ROP of Alanine N-carboxyanhydride (NCA-ALA) was conducted by following procedure described in lietrature.[1] Briefly stated, 931 mg NCA-Ala (8.1 mmol) was mixed in 20 mL DMSO solvent in a flame-dried reaction flask equipped with a stirring bar and mechanically stirred for 1 h to get a well-dispersed solution. The solution was kept in an ice bath to maintain 0 ºC, followed by adding 56 µL propane-1,3-diamine (0.67 mmol) into it under the constant flow of nitrogen (N_2) gas. Reaction was continued for 5 days at room temperature (RT) in an inert N_2 atmosphere, described in **Scheme S1**. A yellowish white solid product was obtained by precipitating the reaction mixture into 5x excess chilled diethyl ether, washed twice with diethyl ether and water, followed by lyophilization. Product **(1)** was characterized by MALDI-TOF Mass Spectrometry (**Figure S1**) after drying overnight at 40 ºC under vacuum.

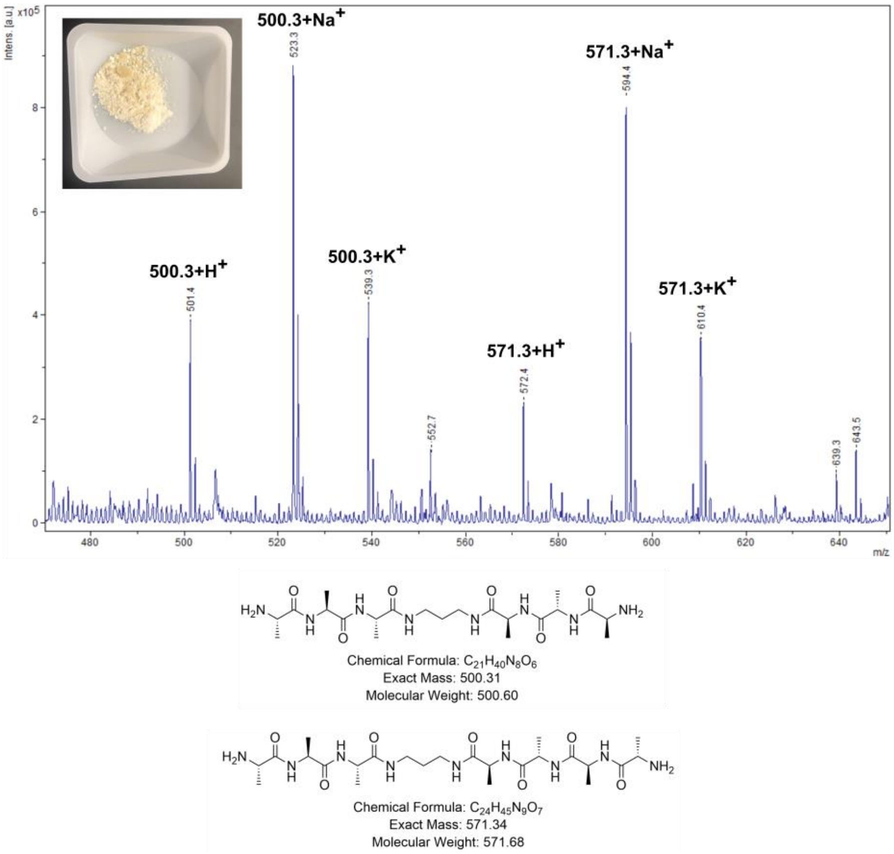

Figure S1. MALDI-TOF MS of oligo(alanine)propandiamine (**1**), appearance of specified masses confirms successful synthesis. Result suggests the formation of polydisperse oligo(alanine)propandiamine **(1)**. The product photograph is inscribed above.

Scheme S2. End group functionalization of oligo(alanine)propandiamine.

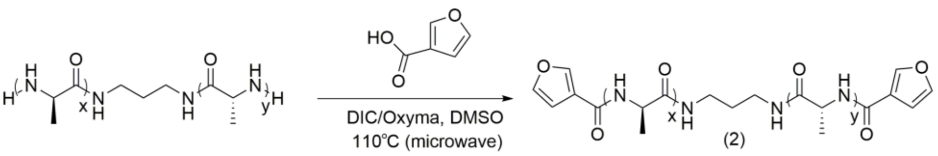

Scheme S2. End-group functionalization: furan derivative of oligo(alanine)propandiamine **(2)**.

oligo(alanine)propandiamine **(1)** was end-group functionalized to furan derivative following **Scheme S2,** a similar procedure described elsewhere.[2] 100 mg oligo(alanine)propandiamine **(1)** (0.175 mmol) and 98 mg 3-furan carboxylic acid (0.875 mmol) were mixed in 1.8 mL DMSO solvent and sonicated for 2 minutes to get a clear solution. The coupling reagents DIC (0.7 mmol, 110 µL) and ethyl cyano(hydroxyimino)acetate or oxyma (0.7 mmol, 98 mg) were

added into the pre-mixed solution and transferred into a Biotage 2 mL microwave reaction vial equipped with a stir bar, capped. The reaction was continued at 110 °C for 15 min in a microwave synthesizer. After the reaction completed, the reaction mixture was cooled to RT, and precipitated in 5x excess chilled diethyl ether. A yellowish white solid residue collected by centrifugation, washed with cold ether and water twice, followed by lyophilization. After vacuum drying at 40 ºC for a day, the product **(2)** was characterized by MALDI-TOF MS (**Figure S2**) and ^{1}H NMR spectroscopy (**Figure S3**).

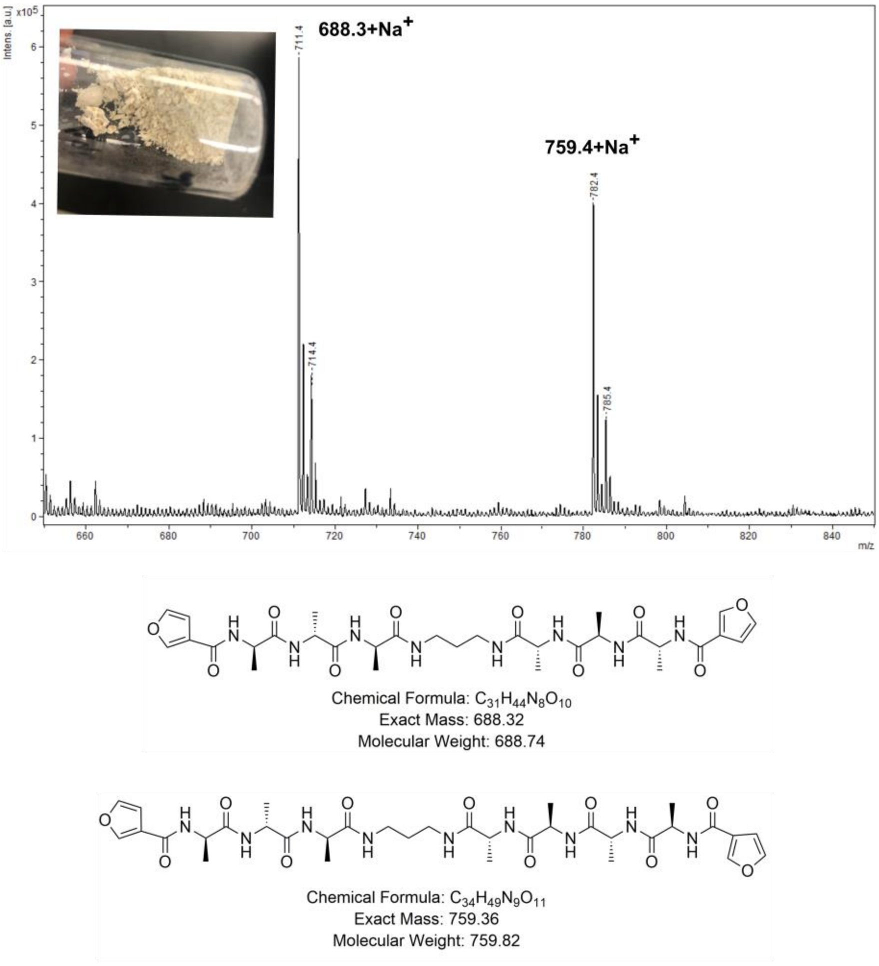

Figure S2. MALDI-TOF MS of furan derivative of oligo(alanine)propandiamine (**2**) demonstrates presence of specified masses, that confirms successful end-group functionalization. The product photograph is inscribed above.

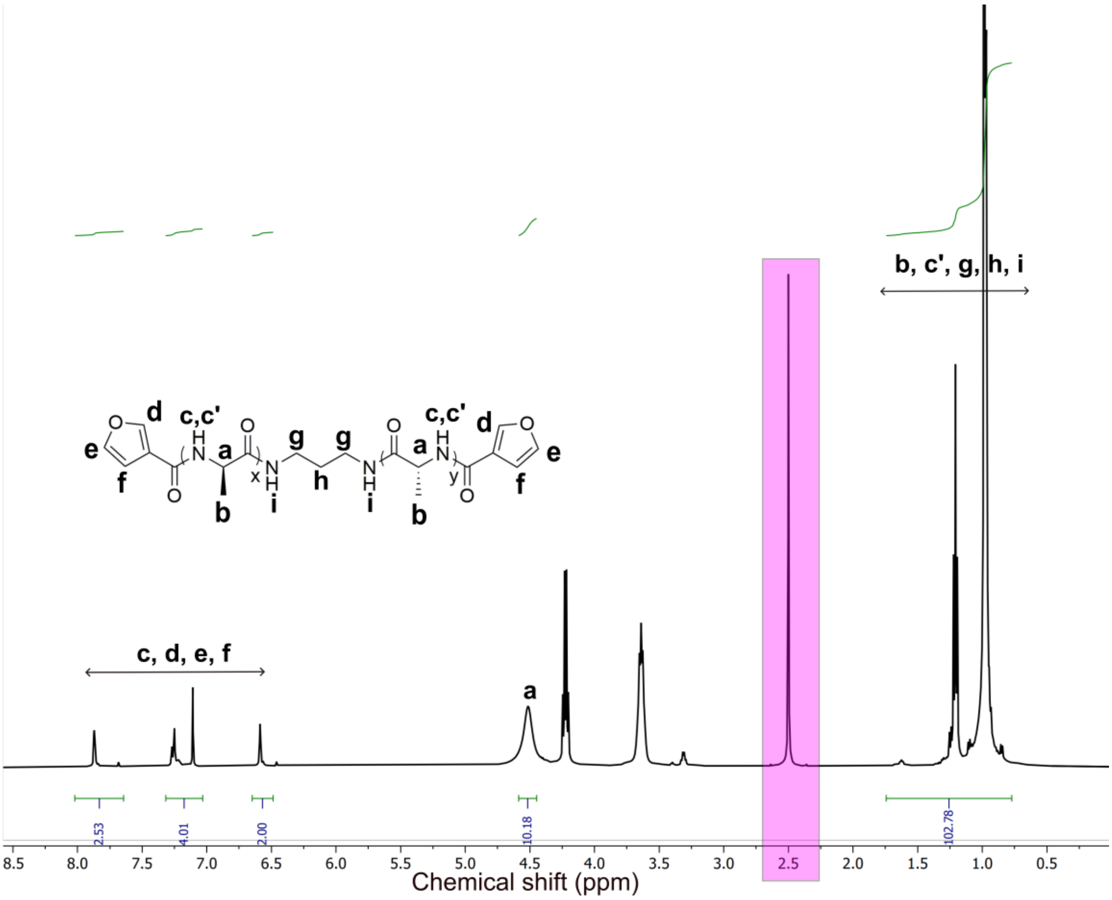

Figure S3. ^{1}H NMR of furan derivative of oligo(alanine)propandiamine (**2**). Spectrum was obtained in DMSO-d6, highlighted in pink. ^{1}H NMR signals from furan moieties appeared in the range of 6.5-8 ppm, that indicates successful incorporation of furan end-group in the oligo(alanine) precursor. The methine (CH) and methyl (CH_3) protons for polyalanine segment appear at 4.5-4.6 and 1.6-1.7 ppm, respectively. Traces of Alanine NCA monomers are found at 4.2 and 1.2 ppm.

Scheme S3. Silk inspired polymer (4) synthesis via Diels-Alder reaction and step-growth polymerization.

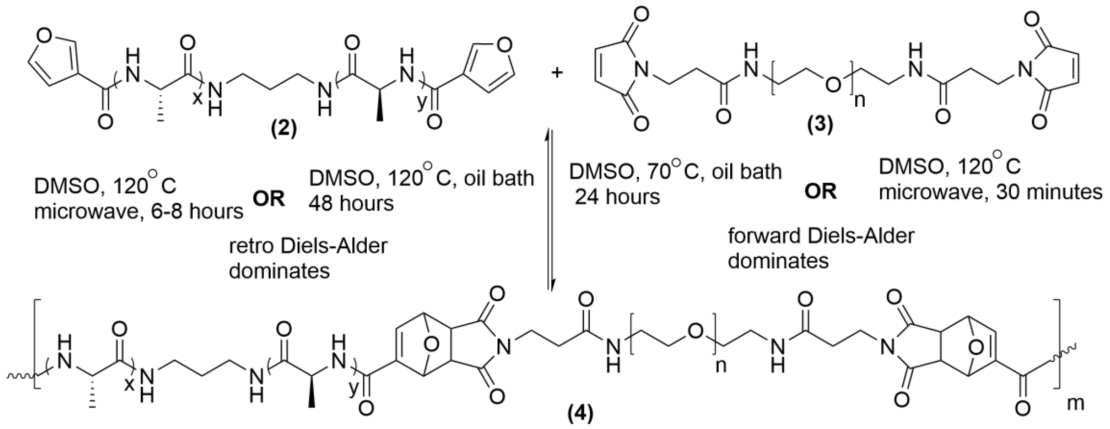

Scheme S3. Silk-inspired polymer **(4)** synthesis by condensation between two moieties via Diels-Alder aided step-growth polymerization.

Silk inspired polymer (**4**) was synthesized ligating furan functionalized oligo(alanine)propandiamine (**2**) and PEG (maleimide)$_2$ **(3)** through a furan-maleimide Diels-Alder (DA) based step-growth polymerization. DA reaction is chosen due to its excellent ability of making covalent bond via one-step process.[3] 100 mg (**2**) (0.145 mmol) was dissolved in 1.5 mL DMSO and sonicated for 2-5 minutes to get a clear solution. Separately, PEG (maleimide)$_2$ (average M_n = 2,000 g/mol, 291 mg, 0.145 mmol) **(3)** dried overnight at 40 °C using schlenk line was dissolved in 0.5 mL DMSO. Next, both were mixed in a Biotage 2 mL microwave reaction vial equipped with a stir bar, capped. Polymerization was continued in a microwave reaction chamber at 120 °C for 30 minutes following **Scheme S3**. After completion, the reaction mixture was cooled to RT, and precipitated in 5x excess chilled diethyl ether. A white solid residue collected by centrifugation and washed with cold ether twice. The product was purified by dialysis against water in a Spectra/Por Float-A-Lyzer G2 dialysis tube of MWCO 500 Da cellulose ester membrane (Spectrum Laboratories) for 3 days. A white solid polymer **(4)** was obtained upon lyophilization and characterized. (**Figure 1**). By changing reaction time to 2 h, resultant polymer **P4** was found with no increment in molecular weight. Likewise, the same reaction condition was repeated in the microwave chamber at 70 ºC for 2 hours that led into

polymer **P5** with the similar M_n and M_w, like polymer **(4)** and **P4**. Similar reaction was repeated with PEG (maleimide)$_2$ **(3)** with average M_n = 5,000 g/mol. Resultant polymer **(P1)** was characterized by GPC (**Figure S4**). Another polymer **P6** was synthesized following the same protocol, just changing the solvent from DMSO to DMF, and found no changes in the molecular weights. Please note that the same polymerization was conducted in an oil bath at relatively lower temperature (70 ºC) for 24 hours, as prolonged reaction at higher temperature (120 ºC) may lead in retro-DA reaction. Resultant polymer **P7** showed similar molecular weight as **P1** synthesized by microwave. These findings and comparative literature research (**Table S2**) suggest that use of microwave not only increases coupling efficiency and decreases polymerization time, but a short-timed reaction at higher temperature may not initiate retro DA reaction. Longer time reaction (**Condition I**: 120 ºC, 6-8 hours in a microwave chamber, product: **P2, Condition II**: 120 ºC, 48 hours in oil bath, product: **P3**) led in retro-DA dominant product. Polymer characterization details are listed in **Table S1**.

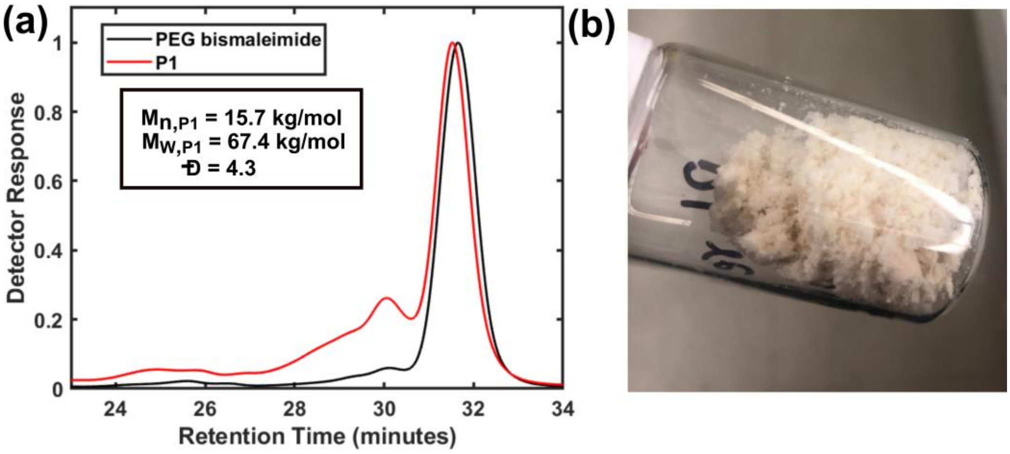

Figure S4. GPC elugram of polymer **P1** with corresponding PEG(maleimide)$_2$5000 homopolymer (a). The shift of the GPC trace to the lower retention time indicates an increase in the molecular weight after the polymerization. The peaks of oligo(alanine)propandiamine and its furan derivative fragments overlap with the solvent peaks (not shown). **P1** polymer photograph is shown in (b).

Synthesized Silk-Inspired Polymers Details

It is important to note that step-growth polymerization must be performed with equal concentrations of both prepolymers, oligo(alanine)propandiamine furan derivative **(2)** and PEG(maleimide)$_2$ **(3).** Failure to do so, it could result into lower molar masses as that causes the deviation from the Carothers equation.[4,5] Since ROP led into polydisperse oligo(alanine)propandiamine **(1)** (**Figure S1**), DA aided step-growth polymerization might deviate from Carother's equation due to incorrect stoichiometry. This could end up with silk-inspired polymers with relatively lower molar masses (**Table S1**).

Table S1. Silk-inspired polymer details.

Entry	Notes	$^{b}M_n$ (kg/mol)	$^{b}M_w$ (kg/mol)	^{b}Dispersity (Đ)
**P1**	PEG bismaleimide5000 & **(2)** were polymerized in DMSO using microwave irradiation at 120 ºC for 30 minutes	15.7	67.4	4.3
**P2**	PEG bismaleimide5000 & **(2)** were polymerized in DMSO using microwave irradiation at 120 ºC for 8 hours.	---	---	---
**P3**	PEG bismaleimide5000 & **(2)** were polymerized in DMSO in an oil bath heated at 120 ºC for 48 hours.	---	---	---
**4**	PEG bismaleimide2000 & **(2)** were polymerized in DMSO using microwave irradiation at 120 ºC for 30 minutes	5.8	12.5	2.2
**P4**	PEG bismaleimide2000 & **(2)** were polymerized in DMSO using microwave irradiation at 120 ºC for 2 h.	5.4	11.1	2.1
**P5**	PEG bismaleimide2000 & **(2)** were polymerized in DMSO using microwave irradiation at 70 ºC for 2 h.	5.7	12.8	2.2
**P6**	PEG bismaleimide5000 & **(2)** were polymerized in DMF using microwave irradiation at 120 ºC for 30 minutes	15.9	66.5	4.2
**P7**	PEG bismaleimide5000 & **(2)** were polymerized in DMSO in an oil bath for 24 hours at 70 ºC	15.5	67.4	4.3
**PEG (maleimide)$_2$ 5000**	Commercially available	11.4	25.1	2.2

PEG (maleimide)$_2$ 2000	Commercially available	4.8	5.3	1.1

[a]polymer molecular weights and dispersity obtained by GPC performed in DMF (0.1 wt% LiBr), calibrated with polymethylmethacrylate (PMMA) standards. Molecular weights were estimated integrating across all polymer peaks, excluding the peaks corresponding to the PEG(maleimide)$_2$ and peptide prepolymers. --- No polymerization occurs.

To analyze the efficiency of our proposed reaction strategy, we compare our result with a few representatives reported linear polymers (**Table S2**), that were synthesized using bifunctional A-A and B-B type macromonomers (e.g., bifuran and bismaleimide) and via furan-maleimide Diels-Alder reaction.

Table S2. Comparison of our proposed microwave-assisted Diels-Alder based polycondensation reaction strategy to other reported work.

Entry	Bifuran structure	Bismaleimide structure	Experimental conditions	M_n (kg/mol)	M_w (kg/mol)	(Đ)	Reference
1	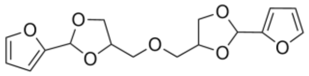	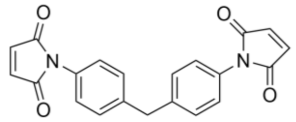	Chloroform ($CHCl_3$), 60ºC, 48 h	2.2[a]	5.4[a]	2.4	6
2	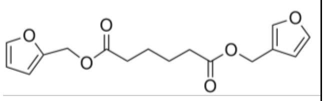	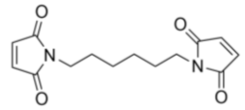	Chloroform ($CHCl_3$), 55 ºC, 48 h	5.9[a]	8.9[a]	1.5	7
3	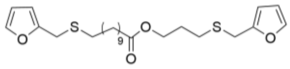	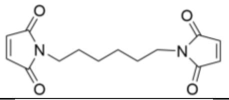	1,1,2,2-tetrachloroethane (TCE), 65 ºC, 8 h	5.8 [a]	9.1 [a]	1.5	8,9
4	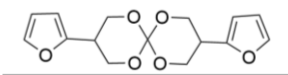	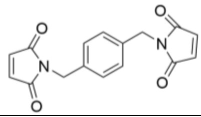	Tetrahydrofuran (THF), reflux, 24 h	---	---	---	10
5	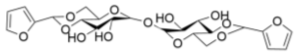	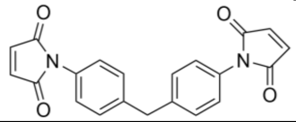	Dimethylformamide (DMF), 70 ºC, 48 h	15.8 [a]	10.8 [a]	1.5	11
6	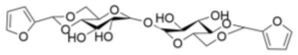	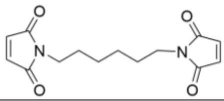	Dimethylformamide (DMF), 55 ºC, 48 h	15.4 [a]	7.6 [a]	2.0	11
7	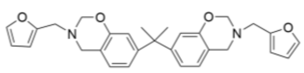	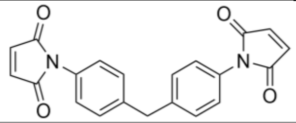	Tetrahydrofuran (THF), 80 ºC, 120 h	19	10.5	1.8	12
8	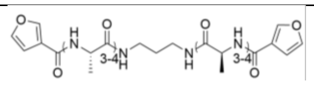	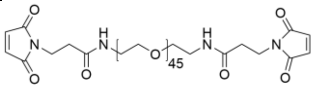	Dimethylsulfoxide (DMSO), 120 ºC, 30 min, microwave	5.8[b]	12.5[b]	2.2	Present work

[a]polymer molecular weight and dispersity obtained by GPC calibrated with polystyrene (PS)[a] or [b]polymethylmethacrylate (PMMA) standards. ----not reported.

Section 2

Thermal analysis.

To study the thermal properties of synthesized homopolymers and silk-inspired polymers, we performed differential scanning calorimetry (DSC) and thermogravimetric analysis (TGA). Our TGA profile indicates that the silk-inspired polymer is thermally stable. Comparative stability analysis along the decomposition temperatures is presented in **Figure 1c** and **Table S3**. Homopolymers oligo(alanine)propandiamine (A block) and PEG (maleilimide)$_2$ 2000 (B block) have distinct thermal characteristics, thus the derived silk-inspired polymer should contain both. We conducted DSC for each candidate, presented in **Figure S5** and **Table S3**. The second heating scans for oligo(alanine)propandiamine (**Figure S5a**) and silk-inspired polymer (**Figure S5c**) both exhibits small peaks at 108 ºC, which is probably due to the residual water elimination from the sample surface, consistent to the TGA result (**Figure 1c**). However, no melting transition was noted for oligo(alanine) below its thermal decomposition temperature, as consistent to other literature.[13] **Figure S5b** shows a typical PEG DSC profile with a melting temperature of 46.7 ºC, which appeared at shifted region of 30.7 ºC in the silk-inspired polymer **(Figure S5c)**. This is consistent with reported work[14] where lower melting point for PEG has been observed in PEG-peptide conjugates. This is attributed to the formation of hydrogen bond between the amide linkage of peptide moieties (alanine in the present study) and the ether linkage of the PEG chain. A T_g value of -52 ºC was observed in the DSC profile for silk-inspired polymer which corresponds to the ether linkage of B block.[1]

Table S3. TGA & DSC data.

Entry	$T_{d,5\%}$ (ºC)	$T_{d,10\%}$ (ºC)	$T_{d,50\%}$ (ºC)	$T_{d,80\%}$ (ºC)	T_g(ºC)	T_m(ºC)
oligo(alanine)propandiamine **(1)**	108	167	351	401	---	---
PEG (maleimide)$_2$ **(3)**	314	352	395	411	---	47
Silk-inspired polymer **(4)**	256	334	395	423	-52	31

$T_{d,5\%}$, temperature of 5% weight loss, $T_{d,10\%}$, temperature of 10% weight loss, $T_{d,50\%}$, temperature of 50% weight loss, $T_{d,80\%}$, temperature of 80% weight loss. Measured by TGA. Tg was determined by the DSC.
--- not observed

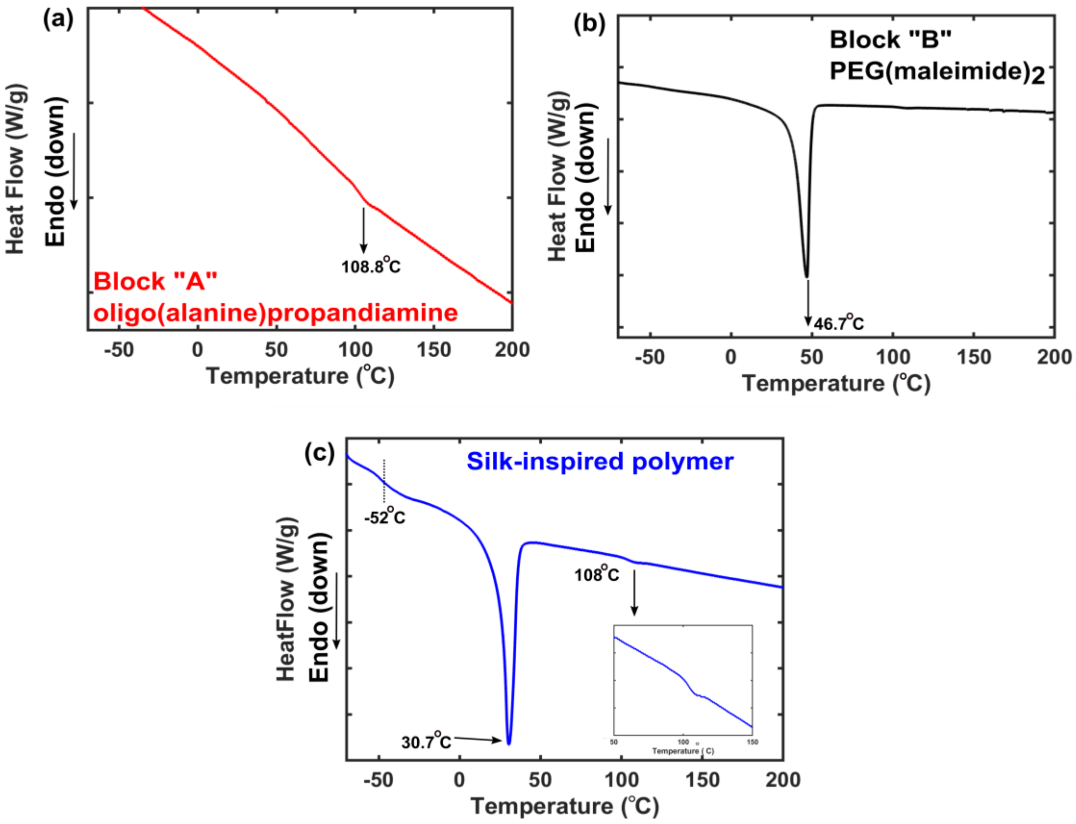

Figure S5. DSC profiles for the homopolymers (a-b) and silk-inspired polymer (c), the second heating ramp is shown.

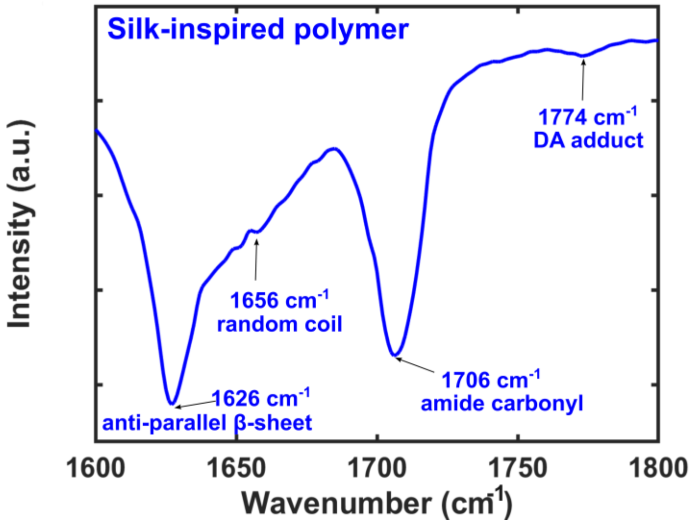

Figure S6. FTIR spectrum of silk-inspired polymer **(4)** along the assigned peaks. Peak at 1774 cm^{-1} (specific to furan-maleimide DA adduct)[15] confirms that DA chemistry does not affect the formation of antiparallel β-sheet structure in the synthesized polymer (evident by the peaks at 1626 cm^{-1}).

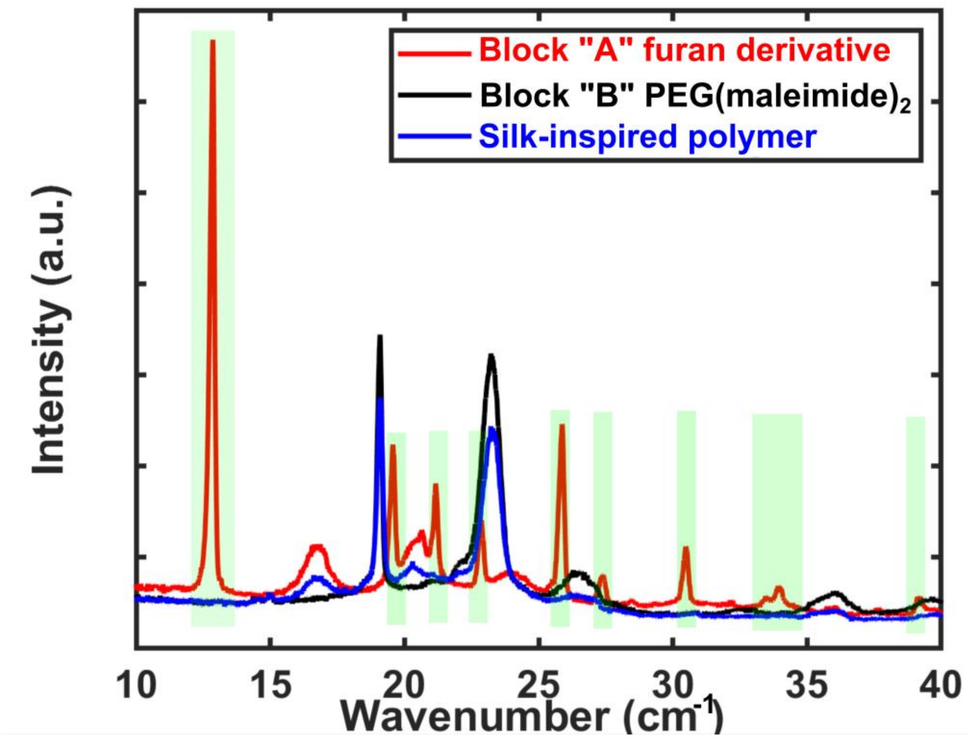

Figure S7. XRD profile for silk-inspired polymer **(4)** demonstrates complete disappearance of furan crystalline peaks (highlighted in green) after DA aided step growth polymerization.

Section 3

Synthesis of Graphene Oxide (GO)

GO was synthesized following procedure by Hummers' method, described elsewhere.[16,17] Synthesis steps are shown in **Figure S8**. Briefly stated, 3 g commercial graphite flakes (vacuum dried at 40 ºC overnight) was added to 100 mL concentrated H_2SO_4 in a three-neck flask under stirring at RT. After that 1.5 g of sodium nitrate was added while the mixture was kept into ice bath, cooled to 0ºC. Under vigorous stirring, 9 g $KMnO_4$ was added slowly (over the span of 30 minutes) and the temperature of the suspension was maintained at 10 ºC. The reaction system was transferred to 35 ºC pre-heated oil bath and stirred for an additional 1 h. During this course a thick brownish grey colored paste was formed along with diminishing in effervescence. 200 mL DI water was added into it slowly which causes violent effervescence. The solution was stirred for an additional 1h at 90 ºC. Next, 300 mL of warm DI water was added slowly into it followed by addition of 20 mL 30% H_2O_2. This turned the color of the solution to golden yellow

from dark brown (**Figure S8a**). The mixture was filtered (while hot) and the filtered solid was centrifuged with 500 mL HCl aqueous solution (1:10) and 500 mL DI water to remove metal ions and the other impurities (**Figure S8b)**. The resultant solid powder was lyophilized to yellowish black powder (**Figure S8c**).

Reduction of Graphite Oxide (GO) via hydrazine monohydrate.

Reduced GO was prepared following published literature.[16,17] 500 mg GO powder was added to 500 mL mixture of DI water and ethanol (1:1 v/v) and sonicated for 15 minutes. The resulting solution turned into yellow color (**Figure S8d**). This sonicated GO solution was mixed with 200 µL hydrazine monohydrate and heated (refluxed) 2 hours at 100 ºC in a pre-heated oil bath. Upon completion the rGO formed agglomerates and floated on the top (**Figure S8e**), filtered, centrifuged/washed with 1 L DI water and lyophilized to obtain black powder (**Figure S8f**). The rGO synthesized via this way precipitates in aqueous medium instantaneously (**Figure S9**) as it becomes hydrophobic due to the elimination of oxygenated functional groups.

Reduction of GO via silk-inspired polymer (4).

500 mg GO powder and 2.5 g silk-inspired polymer (GO:polymer weight ratio was maintained to 1:5) were dispersed to 500 mL 1M NaOH mixture of DI water and ethanol (1:1 v/v) and mechanically stirred for 15 minutes. Final solution pH was maintained to ~12 to avoid polymer aggregation and reducing the time of reaction.[18-20] Reduction was continued at 40 ºC on a pre-heated oil bath for 2 hours. Distinctively, no black precipitation was found, rather dispersed/suspended in the liquid media (**Figure S8g**). It is attributed to the stabilization effect of the polymer **(4)**. Typically, rGO becomes hydrophobic due to the removal of oxygenated functional groups and thus precipitates. However, the polymer coats the rGO surface here and increases its compatibility to the aquatic environment. This observation suggests that our designed silk-inspired polymer not only acts as a reducing agent, but also plays a lead role in stabilizing rGO in aqueous media. The resulting black powder was filtered, centrifuged with 1 L DI water and redispersed in ddH_2O three times to ensure the successful removal of the free

unbound polymer. Finally, it was lyophilized to obtain black rGO powder (**Figure S8h**). The same reduction experiment was repeated with a modified GO:polymer weight ratio of 1:1 (500 mg polymer was added to the 500 mg GO aqueous solution). Despite successful reduction, we did not achieve the stable dispersion of rGO like earlier condition, rather it precipitates by forming aggregates (**Figure S9**). It indicates that a larger weight ratio of polymer/GO is needed to achieve both reduction and stabilization effect. This observation is consistent with the earlier report on glycine reduced GO.[21] Please note that all the characterizations (FTIR, XRD, SEM. BET, etc.) were performed with the rGO obtained with polymer:GO weight ratio of 1:1.

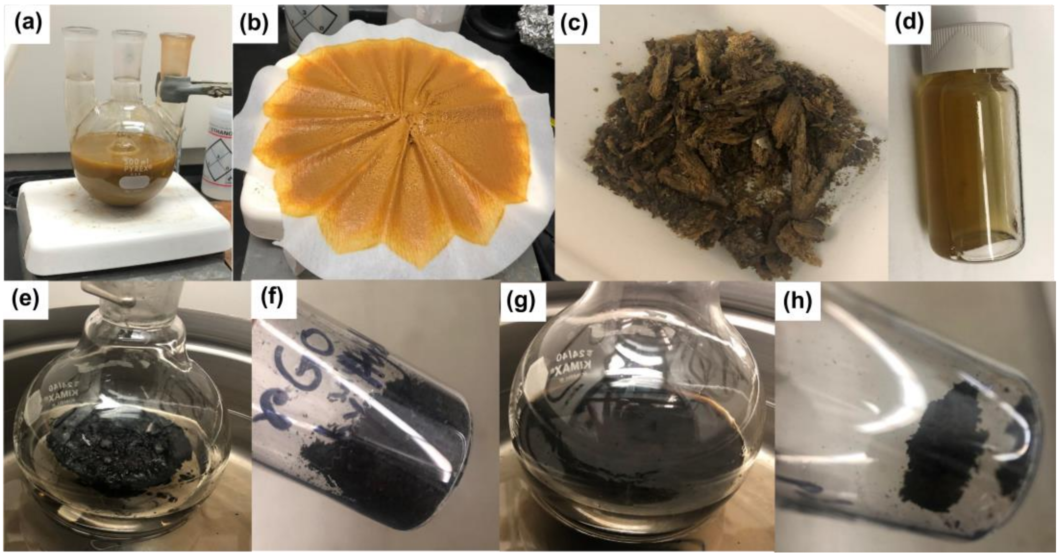

Figure S8. Experimental procedure of GO (a-b) results in yellowish powder (c). 15 minutes sonication of GO in ethanol/water mixture results in exfoliation of GO that turns the solution into yellow color (d). Upon reduction by hydrazine monohydrate, the yellow dispersed solution turns into black aggregate of rGO (e), as the π-π and hydrophobic interactions dominate in rGO sheets. Lyophilized black rGO powder is shown in (f). The same reduction was continued using silk-inspired polymer (g) that results in formation of rGO (h), however no precipitation or agglomeration was found (constantly monitored for a day) as the polymer helps to disperse the rGO in water/ethanol medium.

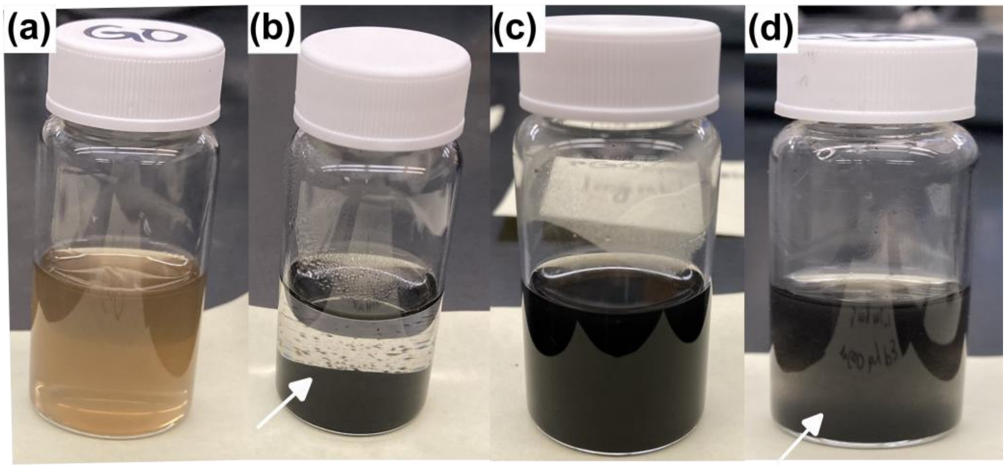

Figure S9. Dispersion stability profiles for graphene derivatives in aqueous media (kept at room temperature for 3 days): aqueous GO (a), rGO obtained by hydrazine monohydrate (b), rGO prepared by silk-inspired polymer (with GO:Polymer weight ratio of 1:5) (c) and rGO by silk-inspired polymer (with GO:Polymer weight ratio of 1:1) (d). Each sample concentration was maintained at 1 mg mL^{-1} and dispersed in ethanol/water (50:50) mixture. Formation of aggregates in hydrazine reduced GO are shown by white arrows (b). Typically, rGO becomes hydrophobic due to the removal of oxygenated functional groups, and thus precipitates quickly. However, the polymer reduced GO (c) shows its stable aquatic dispersion for 3 days, additionally confirmed by TEM image (**Figure S10**). This observation suggests that our designed silk-inspired polymer not only acts as a reducing agent, but it also plays a role in stabilizing rGO in aquatic media. However, we did not achieve the stable dispersion of rGO in (d), which indicates that larger weight ratio of polymer/GO is needed to achieve stable dispersion.

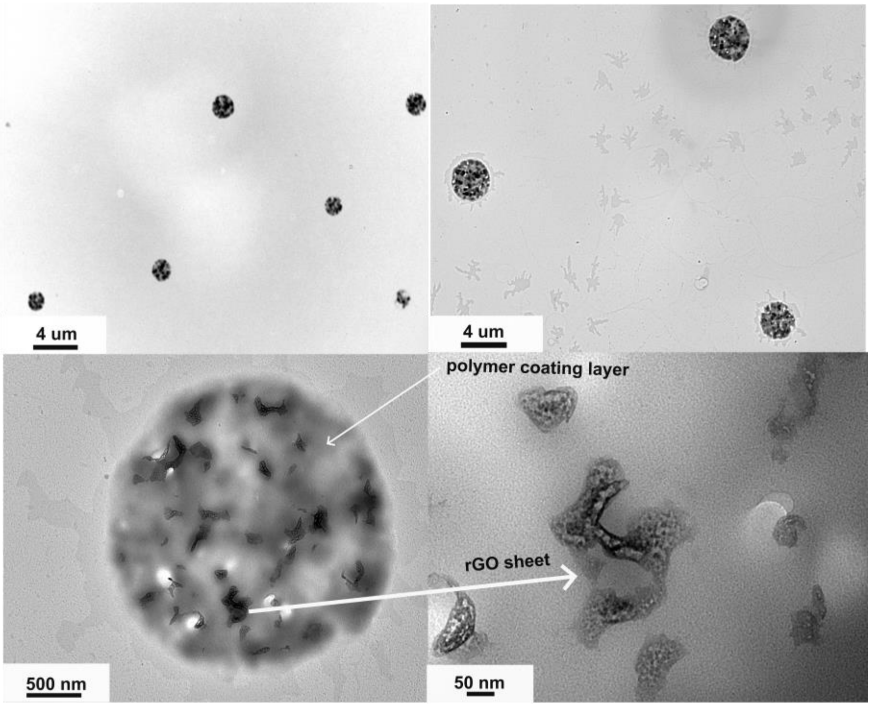

Figure S10. TEM of stable aquatic dispersion of rGO after 3 days (**Figure S9c**) showed that non-aggregated hydrophobic rGO sheets are coated with silk-inspired polymer, and thus suspended in water/ethanol mixture for prolonged time.

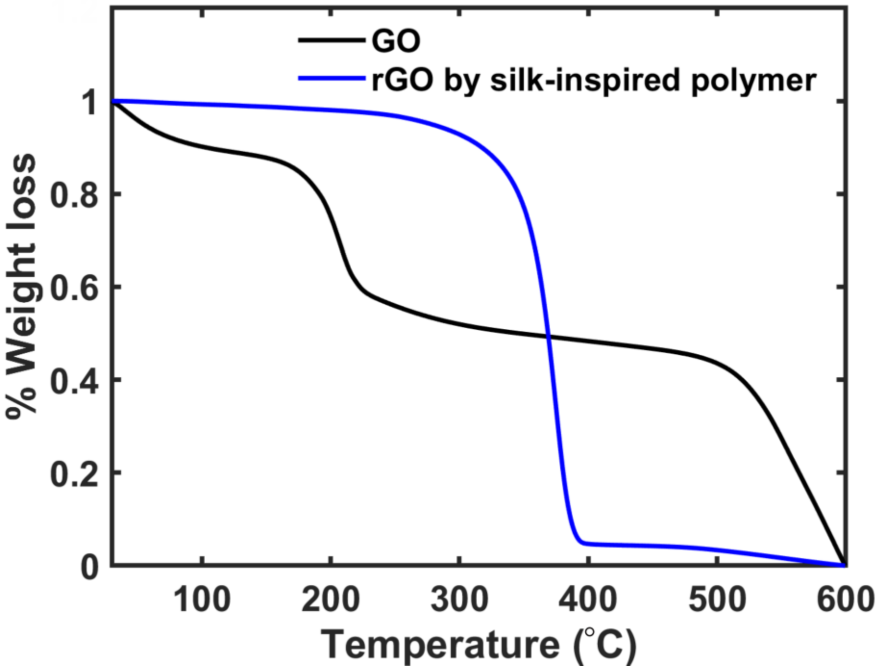

Figure S11. TGA of exfoliated GO (black) and rGO obtained by silk-inspired polymer **(4)** assisted reduction process (blue). For GO two sharp mass losses (15% and 45% decomposition) occured at 170 ºC and 480 ºC, as the oxygenated functional groups were decomposed along

carbon oxidation, respectively. Whereas the polymer assisted rGO showed mass loss, starting at an onset temperature of 335 ºC, illustrating (i) removal of majority oxygenated functional groups upon reduction along (ii) the degradation of bounded residual polymer **(4).**

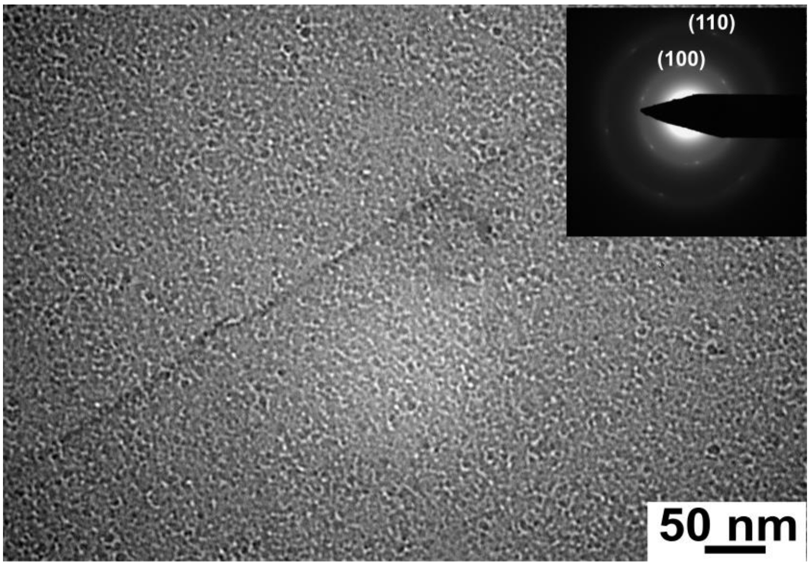

Figure S12. TEM and corresponding SAED pattern with a diffraction ring, but with unresolved diffraction spots for a GO sheet demonstrates its amorphous nature.

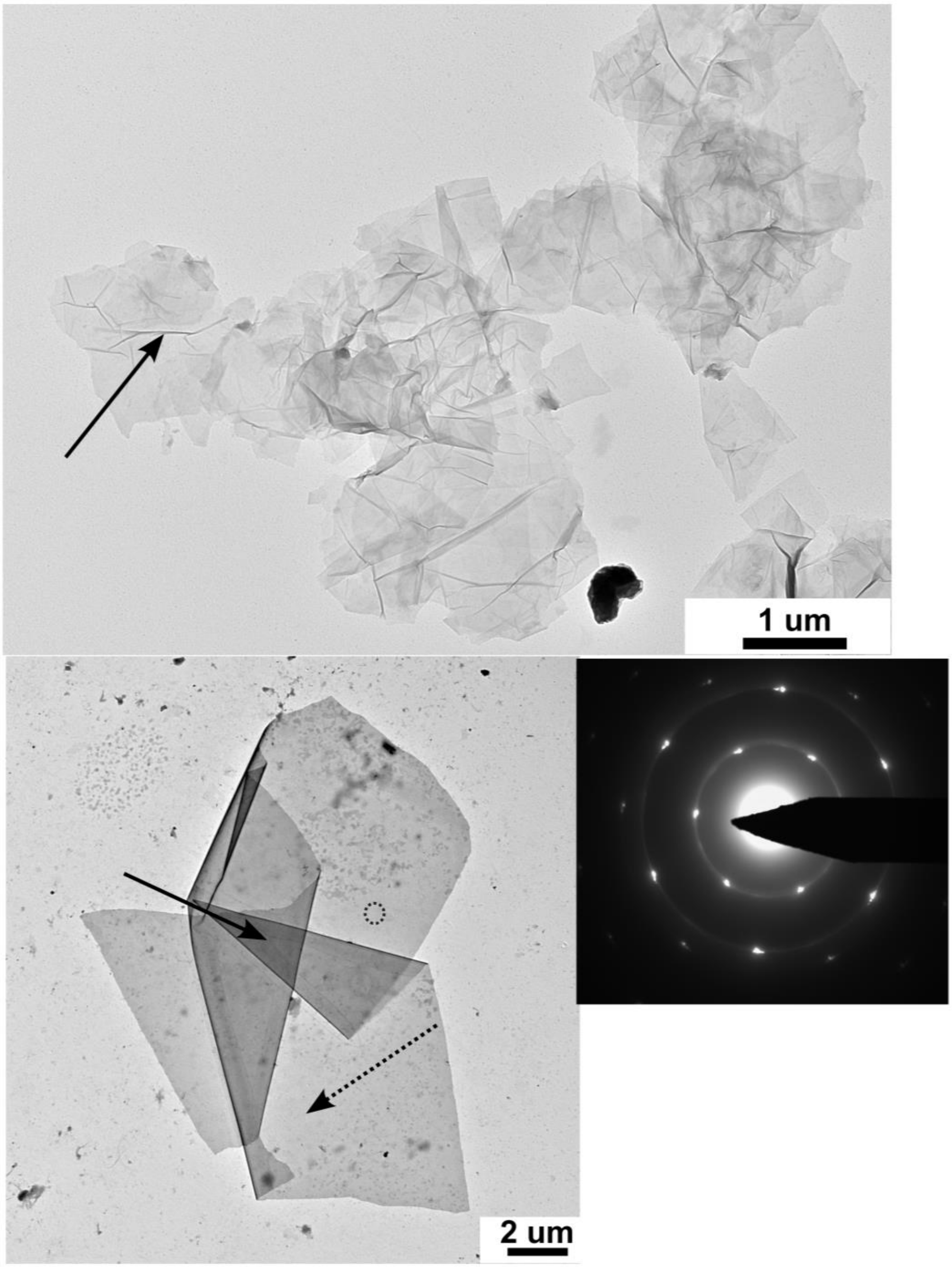

Figure S13. TEM image of suspended rGO sheets (**Figure S9c**) suggesting monolayer (homogeneous layer shown by dotted arrow) prepared by silk-inspired polymer assisted reduction method. Folded regions in the rGO sheets are shown by black arrow (dark region). SAED analysis for the selected area (dotted circle) showed well-defined diffraction spots of six-fold symmetry and confirmed the presence of ordered graphite crystal structures in graphene sheets.

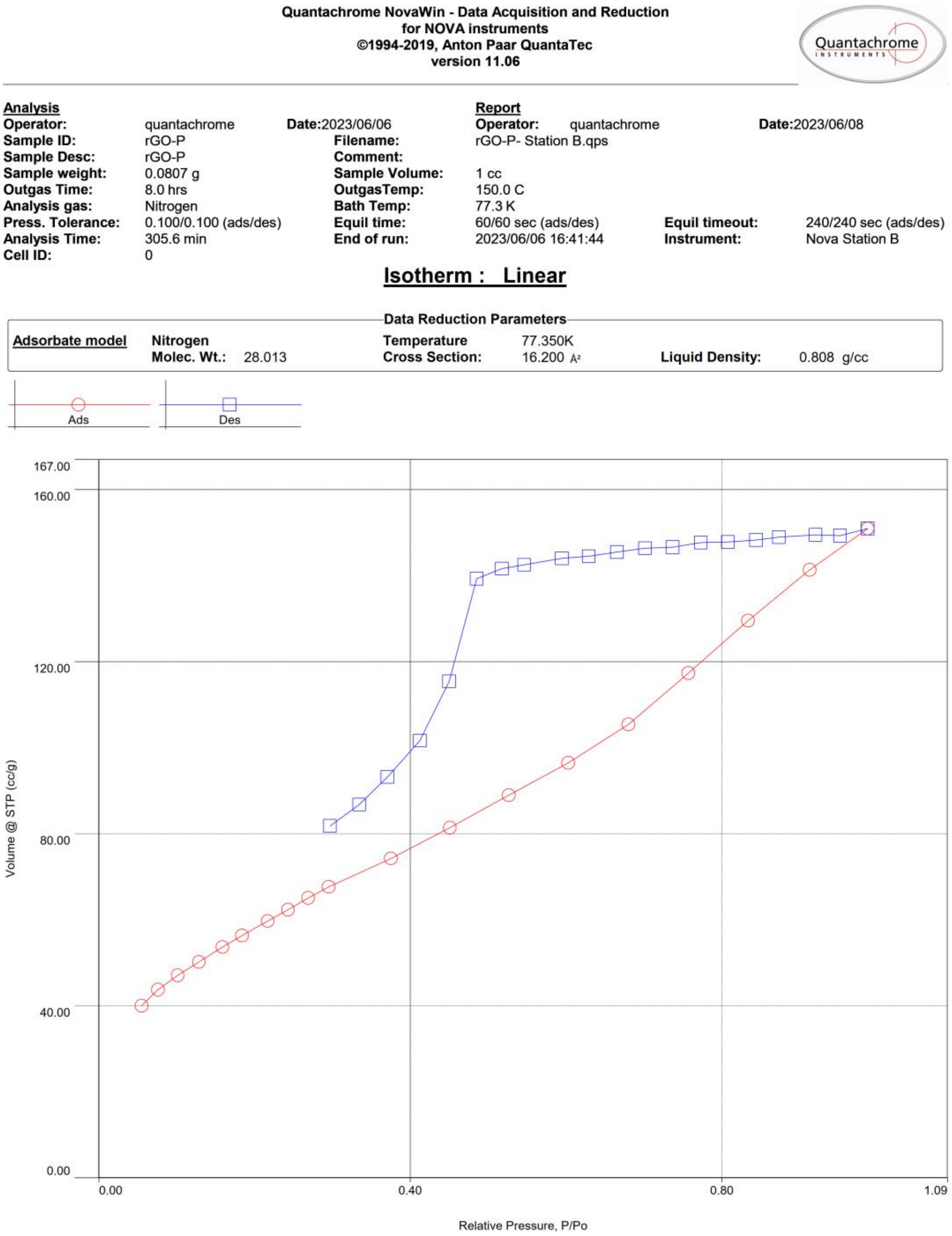

Figure S14. Nitrogen adsorption and desorption isotherms for rGO prepared by silk-inspired polymer assisted reduction method.

Table S4. Comparison of the BET surface area of the current rGO to the other published work.

Entry	Reduction method/sample type	Surface area m^2/g	Pore size (nm)	Reference
1	Single Graphene sheet	2620	---	16
2	Hydrazine hydrate (N_2H_4, H_2O), 100 ºC, 24 hours	466	Both micro and mesopores	16
3	Hydrazine monohydrate, 80 ºC, 12 hours	82-487	---	17
4	Sodium borohydride ($NaBH_4$), 80 ºC, 10 hours	66	---	22
5	$NaBH_4$, 80 ºC, 2 hours	139.7	---	23
6	Silk-inspired polymer assisted reduction method	217.6	Primarily mesoporous	Present work

--- not reported

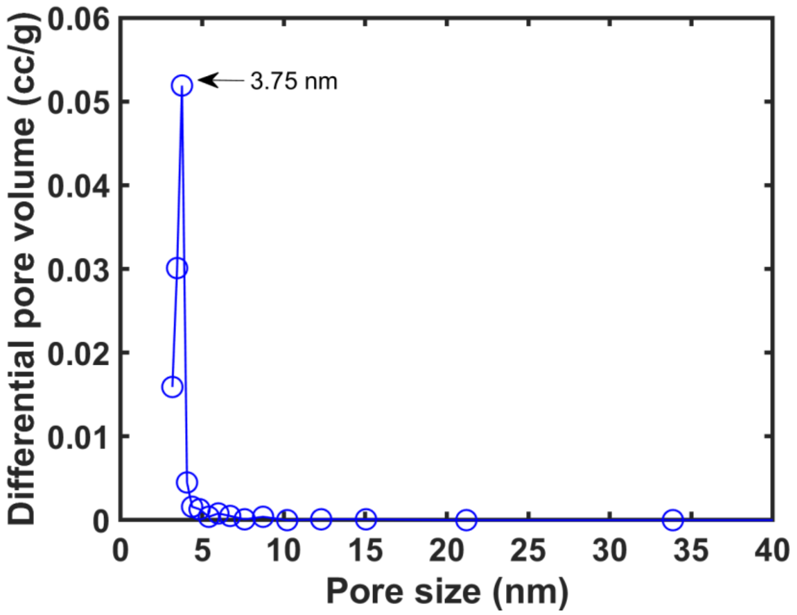

Figure S15. BJH pore size distribution for the rGO prepared by silk-inspired polymer assisted reduction method.

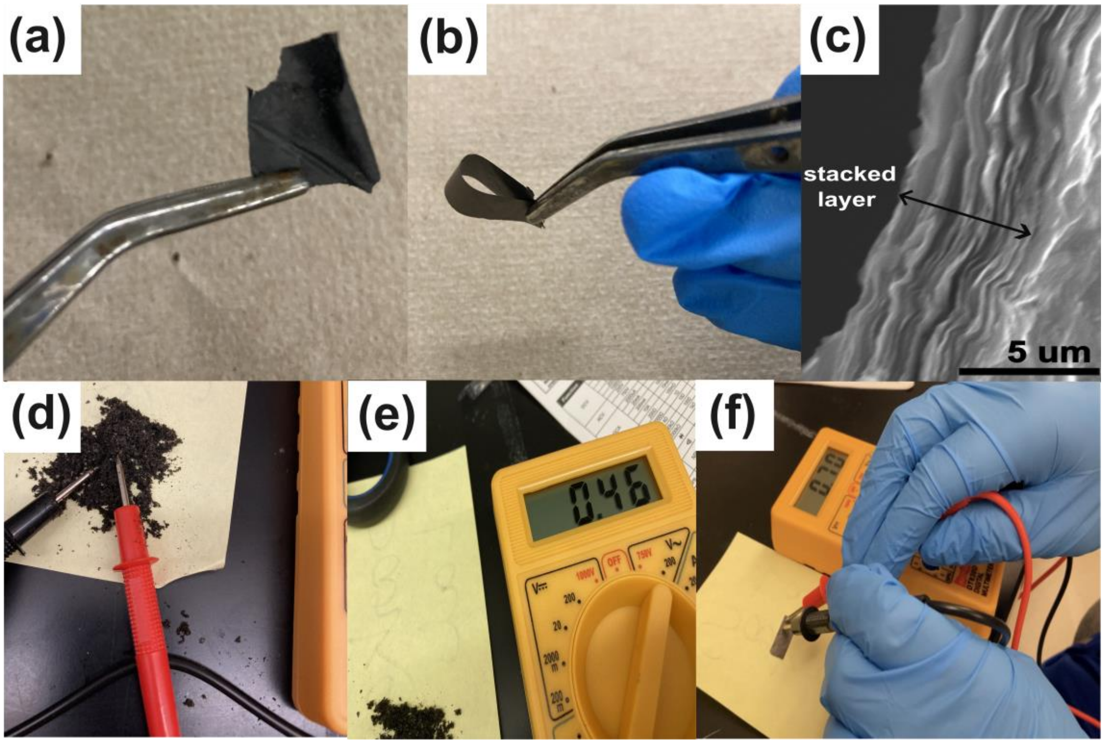

Figure S16. Photograph of our developed rGO free-standing (a) and flexible (b) film/paper like solid material that demonstrates layered structure (c), shown by SEM cross-section image. The electrical conductivity of the rGO powder (d-e) and the rGO film (f) was estimated using a multimeter. The resistivity test for both the powder and film showed that they possess electrical conductivity.

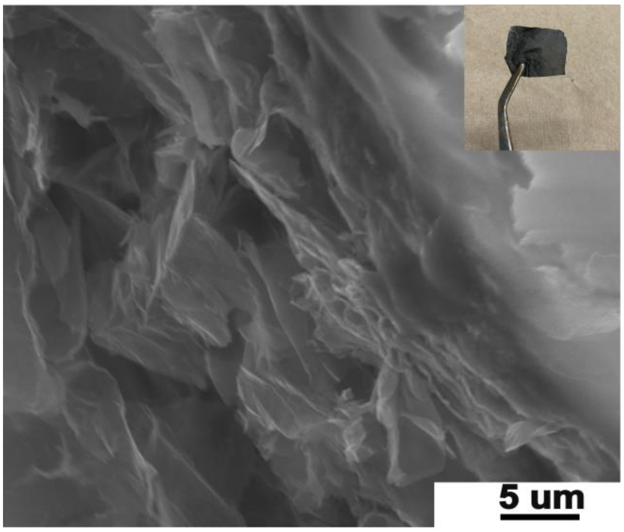

Figure S17. FESEM image of the rGO sheets (obtained by the silk-inspired polymer assisted reduction method) displayed large micrometer-sized crystalline sheets, attractive for electronic applications.

References

1. Rathore, O.; Sogah, D. Y. Self-Assembly of β-Sheets into Nanostructures by Poly(alanine) Segments Incorporated in Multiblock Copolymers Inspired by Spider Silk. *J. Am. Chem. Soc.* **2001**, *123*, 5231-5239.

2. Sarkar, A.; Edson, C.; Tian, D.; Fink, T. D.; Cianciotti, K.; Gross, R. A.; Bae, C.; Zha, R. H. Rapid Synthesis of Silk-Like Polymers Facilitated by Microwave Irradiation and Click Chemistry. *Biomacromolecules* **2021**, *22,* 95-105.

3. Maleki, A.; Taheri-Ledari, R.; Soroushnejad, M. Surface Functionalization of Magnetic Nanoparticles via Palladium-Catalyzed Diels-Alder Approach. *ChemistrySelect* **2018**, *3*, 13057-13062.

4. Zhang, L.; Ren, X.; Zhang, Y.; Zhang, K. Step-Growth Polymerization Method for Ultrahigh Molecular Weight Polymers. *ACS Macro Lett.* **2019**, *8,* 948-954.

5. Estupinan, D.; Gegenhuber, T.; Blinco, J. P.; Barner-Kowollik, C.; Barner, L. Self-Reporting Fluorescent Step-Growth RAFT Polymers Based on Nitrile Imine-Mediated Tetrazole-ene Cylcoaddition Chemistry. *ACS Macro Lett.* **2017**, *6*, 229-234.

6. Satoh, H.; Mineshima, A.; Nakamura, T.; Teramoto, N.; Shibata, M. Thermo-reversible Diels-Alder Polymerization of Difurfurylidene Diglycerol and Bismaleimide. *React. Funct. Polym*. **2014**, *76*, 49-56.

7. Canadell, J.; Fischer, H.; De With, G.; van Benthem, R. A. T. M. Stereoisomeric Effects in Thermo-remendable Polymer Networks Based on Diels-Alder Crosslink Reactions. *J. Polym. Sci. Part A Polym. Chem*. **2010**, *48,* 3456-3467.

8. Gandini, A.; Carvalho, A. J. F.; Trovatti, E.; Kramer, R. K.; Lacerda, T. M. Macromolecular Materials Based on the Application of the Diels-Alder Reaction to Natural Polymers and Plant Oils. *Eur. J. Lipid Sci. Technol*. **2018**, *120*, 1700091.

9. Vilela, C.; Cruciani, L.; Silvestre, A. J. D.; Gandini, A. Reversible Polymerization of Novel Monomers Bearing Furan and Plant Oil Moieties: A Double Click Exploitation of Renewable Resources. *RSC Advances* **2012**, *2*, 2966-2974.

10. Gandini, A. The Furan/Maleimide Diels-Alder Reaction: A Versatile Click-Unclick Tool in Macromolecular Synthesis. *Prog. Polym. Sci*. **2013**, *38*, 1-29.

11. Teramoto, N.; Arai, Y.; Shibata, M. Thermo-Reversible Diels-Alder Polymerization of Difurfurylidene Trehalose and Bismaleimides. *Carbohydrate Polymers* **2006**, *64*, 78-84.

12. Chou, C. I.; Liu, Y. L. High Performance Thermosets from A Curable Diels-Alder Polymer Processing Benzoxazine Groups in the Main Chain. *J. Polym. Sci. Part A Polym. Chem*. **2008**, *46*, 6509-6517.

13. Yu, H.; Kalutantirige, F. C.; Yao, L.; Schroeder, C. M.; Chen, Q.; Moore, J. S. Self-Assembly of Repetitive Segment and Random Segment Polymer Architectures. *ACS Macro Lett.* **2022**, *11*, 1366-1372.

14. Castelletto, V.; Newby, G. E.; Zhu, Z.; Hamley, I. W.; Noirez, L. Self-Assembly of PEGylated Peptide Conjugates Containing a Modified Amyloid β-Peptide Fragment. *Langmuir* **2010**, *26*, 9986-9996.

15. Zhang, G.; Zhao, Q.; Yang, L.; Zou, W.; Xi, X.; Xie, T. Exploring Dynamic Equilibrium of Diels-Alder Reaction for Solid State Plasticity in Remoldable Shape Memory Polymer Network. *ACS Macro Lett.* **2016**, *5*, 805-808.

16. Stankovich, S.; Dikin, D. A.; Piner, R. D.; Kohlhaas, K. A.; Kleinhammes, A.; Jia, Y.; Wu, Y.; Nguyen, S. T.; Ruoff, R. S. Synthesis of Graphene Based Nanosheets via Chemical Reduction of Exfoliated Graphite Oxide. *Carbon* **2007**, *45*, 1558-1565.

17. Park, S.; An, J.; Potts, J. R.; Velamakanni, A.; Murali, S.; Ruoff, R. S. Hydrazine-Reduction of Graphite-and Graphene Oxide. *Carbon* **2011**, *49*, 3019-3023.

18. Xu, S.; Yong, L.; Wu, P. One-Pot, Green, Rapid Synthesis of Flowerlike Gold Nanoparticles/Reduced Graphene Oxide Composite with Regenerated Silk Fibroin as Efficient Oxygen Reduction Electrocatalysis. *ACS Appl. Mater. Interfaces* **2013**, *5*, 654-662.

19. Nilogal, P.; Uppine, G. B.; Rayaraddi, R.; Sanjeevappa, H. K.; Martis, L. J.; Narayana, B.; Yallappa, S. Conductive *In Situ* Reduced Graphene Oxide-Silk Fibroin Bionanocomposites. *ACS Omega* **2021**, *6*, 12995-13007.

20. Aunkor, M. T. H.; Mahbubul, I. M.; Saidur, R.; Metselaar, H. S. C. The Green Reduction of Graphene Oxide. *RSC Adv.* **2016**, *6*, 27807-27828.

21. Bose, S.; Kulia, T.; Mishra, A. K.; Kim, N. H.; Lee, J. H. Dual Role of Glycine as a Chemical Functionalizer and a Reducing Agent in the Preparation of Graphene: An Environmentally Friendly Method. *J. Mater. Chem.* **2012**, *22*, 9696-9703.

22. Zhang, W.; Li, Y.; Peng, S. Facile Synthesis of Graphene Sponge from Graphene Oxide for Efficient Dye-Sensitized H_2 Evolution. *ACS Appl. Mater. Interfaces* **2016**, *8*, 15187-15195.

23. Chen, X.; Chen, B. Macroscopic and Spectroscopic Investigations of the Adsorption of Nitroaromatic Compounds on Graphene Oxide, Reduced Graphene Oxide, and Graphene Nanosheets. *Environ. Sci. Technol.* **2015**, *49*, 6181-6189.
